# Computer Simulations of EMHD Casson Nanofluid Flow of Blood through an Irregular Stenotic Permeable Artery: Application of Koo-Kleinstreuer-Li Correlations

**DOI:** 10.3390/nano13040652

**Published:** 2023-02-07

**Authors:** Rishu Gandhi, Bhupendra Kumar Sharma, Nidhish Kumar Mishra, Qasem M. Al-Mdallal

**Affiliations:** 1 Department of Mathematics, Birla Institute of Technology and Science, Pilani 333031, India; 2 Department of Basic Science, College of Science and Theoretical Studies, Saudi Electronic University, Riyadh 11673, Saudi Arabia; 3 Department of Mathematical Sciences, College of Science, UAE University, Al-Ain P.O. Box 17551, United Arab Emirates

**Keywords:** KKL correlations, Casson fluid, stenosis and aneursym, nanoparticles, radiation

## Abstract

A novel analysis of the electromagnetohydrodynamic (EMHD) non-Newtonian nanofluid blood flow incorporating CuO and Al${}_{2}$O${}_{3}$ nanoparticles through a permeable walled diseased artery having irregular stenosis and an aneurysm is analyzed in this paper. The non-Newtonian behavior of blood flow is addressed by the Casson fluid model. The effective viscosity and thermal conductivity of nanofluids are calculated using the Koo-Kleinstreuer-Li model, which takes into account the Brownian motion of nanoparticles. The mild stenosis approximation is employed to reduce the bi-directional flow of blood to uni-directional. The blood flow is influenced by an electric field along with a magnetic field perpendicular to the blood flow. The governing mathematical equations are solved using Crank-Nicolson finite difference approach. The model has been developed and validated by comparing the current results to previously published benchmarks that are peculiar to this study. The results are utilized to investigate the impact of physical factors on momentum diffusion and heat transfer. The Nusselt number escalates with increasing CuO nanoparticle diameter and diminishing the diameter of Al${}_{2}$O${}_{3}$ nanoparticles. The relative % variation in Nusselt number enhances with Magnetic number, whereas a declining trend is obtained for the electric field parameter. The present study’s findings may be helpful in the diagnosis of hemodynamic abnormalities and the fields of nano-hemodynamics, nano-pharmacology, drug delivery, tissue regeneration, wound healing, and blood purification systems.

## 1. Introduction

The World Health Organization (WHO) reports that 68 percent of deaths are caused by non-communicable diseases, of which cardiovascular disorders account for one-third. According to evidence from various physiological studies, disorders of the blood arteries and the heart, such as heart attacks and strokes, are the major cause of mortality globally. Atherosclerosis, a condition that results in plaque building up in the artery lumen and manifesting as stenosis, causes these events because it prevents blood from reaching distant body cells. An aneurysm may be caused by multiple factors that result in the breaking down of the well-organized structural components (proteins) of the aortic wall that provide support and stabilize the wall. The exact cause has yet to be fully discovered. Atherosclerosis is thought to play an essential role in aneurysmal disease. Numerous researchers ([1,2,3,4,5,6,7]) have theoretically and experimentally explored the mechanics of blood circulation via stenosed arteries. The physical characteristics of EMHD of the bloodstream through an artery in the presence of electroosmotic forces with both aneurysm and stenosis are theoretically investigated by Abdesalam et al. [8]. Zhang et al. [9] studied the impacts of nanoparticle volume fraction on plaque disintegration during transit by employing a two-phase mixing approach. By assuming that blood viscosity is hematocrit-dependent, Poonam et al. [10] analyzed the impact of hybrid nanoparticles on hemodynamical blood flow parameters via a curved artery with aneurysm and stenosis. Basha et al. [11] examined the fluid transport behavior of Au-Cu/Blood hybrid nanofluid via an artery having the inclination and irregular stenosis. Using computational fluid dynamics (CFD) in COMSOL Multiphysics, Waqas et al. [12] investigated the numerical modeling of hybrid nanofluid with nanoparticles such as gold and silver over a stenotic artery.

A novel class of functional fluids known as ”nanofluids” has been produced by the colloidal combination of these nanoparticles in the conventional base fluid, having less effective thermophysical properties. As a result, the nanofluid generated has increased thermal conductivity, thermal diffusivity, viscosity, and convective heat transfer coefficient, among other thermophysical properties. These nanoparticles, or nanofluids, have been used strategically in numerous heat transfer applications with outstanding success. However, more biomedical engineering applications, such as drug transport, tissue regeneration, wound healing, and biomagnetic nano-pharmacodynamics, are beginning to emerge. Badfar et al. [13] used Fe${}_{3}$O${}_{4}$ nanoparticles coated with a drug to carry out the magnetic drug targeting in the vessel’s stenosis region. They also investigated how the wire’s location as a magnetic source affected the MDT. In their numerical simulation of magnetic nanoparticle-based medication delivery, Varmazyar et al. [14] used two cases: one with a slight obstruction and the other with two mild and severe blockages. Ramadan et al. [15] analyzed the blood flow incorporating gold nanoparticles via a stenosed tapering artery using the Phan-Thien-Tanner fluid model to treat the terrible cancer disease. In order to accurately characterize the blood flow, including TiO${}_{2}$ and Ag nanoparticles, Saeed et al. [16] used a couple-stress fluid model. Sharma et al. [17] carried out entropy analysis for blood flow with temperature-dependent viscosity through a tapered artery having multiple-stenosis incorporating hybrid nanoparticles. Mekheimer et al. [18] used biomedical models of drug distribution via nanoparticles to address the issue of synovitis in sick tissues. Khanduri et al. [19] investigated the influence of Hall and ion slips on MHD blood flow through a catheterized artery with multiple stenosis and thrombosis while suspending Au and GO nanoparticles in the blood. Sharma et al. [20] analyzed the effects of heat transfer and body acceleration on unsteady MHD blood flow through a curved artery in the presence of stenosis and aneurysm using hybrid nanoparticles. Dolui et al. [21] investigated the combined influence of non-linear thermal radiation and externally induced magnetic field to graphically evaluate the flow characteristics of tri-hybrid (Cu–Ag–Au), hybrid (Cu–Au), and single (Au) nanofluids flowing through arteries with composite stenosis. Karmakar et al. [22] formulated a mathematical framework for the hemodynamical characterization of blood circulation containing trihybrid nanoparticles inside an eccentric endoscopic artery canal with a flexible wall in the presence of buoyancy and electro-osmotic pressures.

The majority of the research described above treated blood as a Newtonian fluid and investigated the relationship between arterial stenosis and blood flow dynamics. The blood behaves in the larger-diameter arteries with an assertive Newtonian behavior when shear rates are greater than 100 s${}^{-1}$. However, because blood is a suspension of cells, it is widely known that arteries with smaller diameters and lower shear rates exhibit remarkable non-Newtonian blood behavior. The Casson fluid flow model has recently become more well-known attributable to its intriguing applications in everyday life. The Casson fluid flow model is widely used in modern science. Casson fluid exhibits yield stress characteristics. The Casson fluid changes into the Newtonian fluid when the yield stress is high enough. Walawender et al. [23] showed that blood can be modeled using Casson fluid by measuring pressure drop and volumetric flow rate experimentally. Sarifuddin et al. [24] used the Marker and Cell approach to numerically solve the equations as they investigated the effect of two-dimensional blood flow while supposing blood to be Casson fluid flowing through an unsteady stenosed artery. Using the Casson model to describe the liquid’s non-Newtonian viscosity, Debnath et al. [25] investigated the effects of a 1st-order homogeneous-heterogeneous chemical reaction in an annular pipe. Darcy’s law was applied by Ali et al. [26] to examine the Casson fluid flow behavior in a 2-D porous channel using a vorticity-stream function method. Das et al. [27] explored solute dispersion via a stenotic tube with an absorptive wall in their study with Casson fluid characterizing the rheology of blood. Padma et al. [28] aimed to investigate how yield stress affected the EMHD motion of Casson fluid and nanoparticles as they flow via a mildly blocked inclined tapering artery.

In their study of blood flow through stenosed arteries, most researchers assumed the artery walls to be impermeable. The primary function of an endothelial wall in the human body is to prevent the exchange of substances between moving tissues and blood. In actuality, the endothelium wall is permeable due to the presence of ultramicroporous structures. Increased permeability is caused by the accumulation of cholesterol, fatty acids, dilated artery walls, and arterial wall injury. Beaver and Joseph’s [29] experimental work revealed that Darcy’s law would not always satisfy the governing equations near the wall. So, they also formulated a boundary condition at the permeable wall. Mishra et al. [30] analyzed the influence of the wall’s permeability via an artery having composite stenosis. Ijaz and Nadeem [31] discussed how the blood flows through a permeable walled stenosed artery is affected by copper nanoparticles. Akbar et al. [32] formulated a mathematical model to simulate blood flow through a composite stenosed artery with permeable walls. Shahzadi and Bilal [33] investigated the flow of blood in a stenosed bifurcated artery containing Copper and its oxide as a drug to reduce the stress and lesions of an atherosclerotic artery. They considered the artery to be permeable as well as compliant. A mathematical model for medication delivery employing gold and alumina nanoparticles via a porous artery with stenosis was developed by Gandhi et al. [34]. In their computational analysis through a curved stenosed permeable artery, Sharma et al. [35] took into account the blood flow in two phases - the core and the plasma region, respectively. Further, Kumawat et al. [36] examined the entropy generation with a chemical reaction through a permeable curved artery. Sharma and Gandhi [37] investigated unsteady heat and mass transmission through a stretching surface immersed in a Darcy-Forchheimer porous medium.

None previously mentioned research assessed the KKL model for the effective thermal conductivity and viscosity models. The primary focus of engineers, physicians, and biologists is on improving the working fluid’s thermal performance. The nanosized particle dispersion in the base fluid is one of the current methods. Now, it is a well-known truth that nanoparticles of one sort or more are trustworthy agents for improving the thermal performance of the working fluid. There are several documented relationships between the thermal characteristics of the base fluid, nanofluid, and solid nanoparticles. One set of relationships has more restrictions than another, though. The role of nanoparticles in drug delivery mechanisms is the most effective technique to cure several problems such as atherosclerosis, aneurysms, angina, or heart attacks. Also, the main concern of physicians during any surgery is to regulate the human body temperature in the presence of external factors such as radiation, heat source, etc. The literature survey reveals that the KKL correlations ([38,39,40]) take temperature, volume fraction, and nanoparticle size into account when calculating how Brownian motion would affect a fluid’s thermal performance. To replicate the nanofluid flow between two parallel plates—one of which is heated from the outside and the other into which coolant fluid is injected, Kandelousi [41] used KKL correlations. Mehmood et al. [42] used the KKL model to assess the effective thermal conductivity and dynamic viscosity of the alumina-water nanofluid in a porous cavity under the influence of an inclined magnetic field. By combining the Cattaneo-Christov flux model with KKL correlations and considering the dispersion of CuO and Al${}_{2}$O${}_{3}$ nanoparticles in blood, Rana and Nawaz [43] examined the improvement of heat transmission. Utilizing the KKL model, Malik et al. [44] thoroughly examined fluid flow between two vertical rotating plates with permeable surfaces, including nanoparticles. Shahzad et al. [45] investigated the behavior of Sutterby nanofluid passing through a sloping sheet considering copper oxide-engine oil (CuO-EO). They determined the effective viscosity and thermal conductivity using the KKL model. Ramzan et al. [46] investigated the heat transmission of MHD water-based nano liquid flow across a permeable stretched curved surface affected by an induced magnetic field by employing KKL correlations for dynamic viscosity and thermal conductivity.

The literature survey shows that no work has been published yet that addresses optimizing heat transfer utilizing the Casson fluid model and KKL correlations along with temperature-dependent viscosity through the irregular stenotic artery with an aneurysm. Therefore, the present study aims to study the blood flow through a stenotic artery with an aneurysm affected by the magnetic field, electric field, Joule heating, radiation, and viscous dissipation. The present work is divided into five main sections: The first part is the introduction concerning KKL correlations and other parameters considered in this work. Secondly, we have the mathematical modeling part, which initially describes KKL correlations for the simulation of nanofluid. Afterward, the model’s geometrical representation and governing equations are presented, followed by non-dimensionalization and radial coordinate transformation. The third section deals with the numerical solution, which firstly discusses the discretization of governing equations and then the validation of the employed Crank-Nicolson finite difference scheme. The validation of the present work is divided into two parts: (i) mesh independence and (ii) validation with existing literature. The following section is the results and graphical analysis, which discusses the influence of various influential parameters involved in the flow. The novelty of the present work is expressed as:

- KKL correlations are employed for modeling nanofluid flow through a permeable stenosed artery.
- EMHD Casson fluid flow is considered along with Joule heating, radiation, and viscous dissipation.
- The relative % variation for the Nusselt number has been calculated and portrayed using bar graphs.

## 2. Mathematical Formulation

An unsteady, incompressible, laminar, viscous, electrically conducting EMHD blood flow through a permeable artery with irregular stenosis and aneurysm is under consideration. A cylindrical coordinate system ($r_{1}^{*}$,$\tilde{\theta }$,$z_{1}^{*}$) is employed with $r_{1}^{*}$ and $z_{1}^{*}$ as radial and axial directions, respectively. The axial symmetry of the artery corresponds to the independence of flow in the azimuthal ($\tilde{\theta }$) direction. The blood behavior is assumed non-Newtonian and is represented using Casson fluid model. The KKL-correlations are used to address variable thermal conductivity and viscosity. A uniform magnetic field $B_{0}$ along with an electric field $E_{0}$ is applied perpendicular to the axial direction ($z_{1}^{*}$-direction) of blood flow. The magnetic Reynold’s number is assumed to be very small ($Re_{M}$≪ 1); therefore, the induced magnetic field is neglected compared to the applied magnetic field.

### 2.1. Mathematical Representation of the Stenosis and Aneursym

The geometry of the stenosis and aneursym is assumed as ([47,48]):

(1) $$R\left(z_{1}^{*}\right)=\left\{\begin{array}{c} R_{0}-2\delta \left[cos\left(\frac{2\pi }{L_{0}}\left(\frac{z_{1}^{*}-d}{2}-\frac{L_{0}}{4}\right)\right)-\frac{7}{100}cos\left(\frac{32\pi }{L_{0}}\left(z_{1}^{*}-d-\frac{L_{0}}{2}\right)\right)\right],\\ \;\;\;\;\;\;\;\;\;\;\;\;\;\;\;\;\;\;\;\;\;\;\;\;\;\;\;\;\;\;\;\;\;\;\;\;\;\;\;\;\;\;\;\;\;\;\;\;\;\;\;\;\;\;\;\;\;\;\;\;\;\;\;\;\;\;\;\;\;\;\;\;\;\;\;\;\;\;\;\;\;\;\;\;\;\;\;\;\;\;\;\;d\leq z_{1}^{*}\leq d+L_{0},\\ R_{0}+2\delta \left[cos\left(\frac{2\pi }{L_{0}}\left(\frac{z_{1}^{*}-d}{2}-\frac{L_{0}}{4}\right)\right)-\frac{7}{100}cos\left(\frac{32\pi }{L_{0}}\left(z_{1}^{*}-d-\frac{L_{0}}{2}\right)\right)\right],\\ \;\;\;\;\;\;\;\;\;\;\;\;\;\;\;\;\;\;\;\;\;\;\;\;\;\;\;\;\;\;\;\;\;\;\;\;\;\;\;\;\;\;\;\;\;\;\;\;\;\;\;\;\;\;\;\;\;\;\;\;\;\;\;\;\;\;\;\;\;\;\;\;\;\;\;\;\;\;\;\;d+2L_{0}\leq z_{1}^{*}\leq d+3L_{0},\\ R_{0},\;\;\;\;\;\;\;\;\;\;\;\;\;\;\;\;\;\;\;\;\;\;\;\;\;\;\;\;\;\;\;\;\;\;\;\;\;\;\;\;\;\;\;\;\;\;\;\;\;\;\;\;\;\;\;\;\;\;\;\;\;\;\;\;\;\;\;\;\;\;\;\;\;\;\;\;\;\;\;\;\;\;\;\;\;\;\;\;\;\mathrm{otherwise}. \end{array}\right.$$

### 2.2. Governing Equations

**Figure 1:**
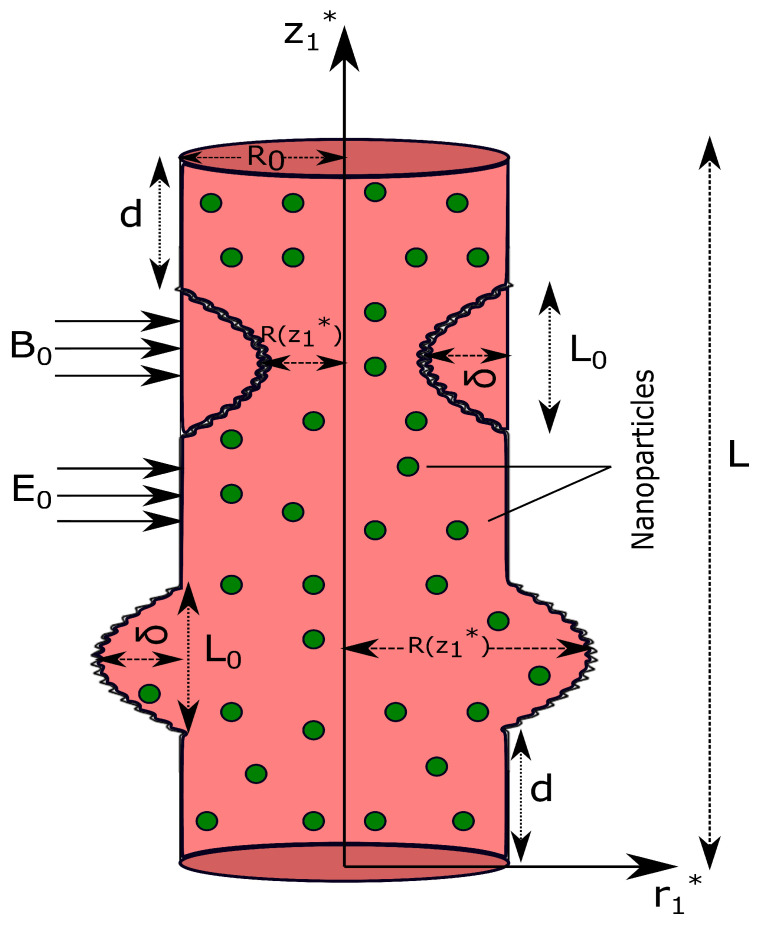
Physical sketch of artery with time variant stenosis and aneurysm.

The velocity and temperature fields are represented as:

$${\tilde{V}}^{*}={\tilde{V}}^{*}\left[u_{1}^{*}\left(r_{1}^{*},z_{1}^{*},t_{1}^{*}\right),\;0,\;w_{1}^{*}\left(r_{1}^{*},z_{1}^{*},t_{1}^{*}\right)\right]\;,\;\;\;\;\;\;{\tilde{T}}^{*}={\tilde{T}}^{*}\left(r_{1}^{*},z_{1}^{*},t_{1}^{*}\right)$$

due to the bidirectional flow of blood through a stenosed artery with aneurysm (Figure 1).

Under the above assumptions and invoking the Boussinesq approximation, the governing equations for the flow are represented as ([34,48]):

**Continuity Equation:**

(2) $$\frac{1}{r_{1}^{*}}\frac{\partial (r_{1}^{*}u_{1}^{*})}{\partial r_{1}^{*}}+\frac{\partial w_{1}^{*}}{\partial z_{1}^{*}}=0,$$

**Momentum Equation:**

$r_{1}^{*}$-direction:

(3) $$\rho_{nf}\left[\frac{Du_{1}^{*}}{Dt}\right]=-\frac{\partial p_{1}^{*}}{\partial r_{1}^{*}}+\frac{1}{r_{1}^{*}}\frac{\partial }{\partial r_{1}^{*}}\left(r_{1}^{*}\tau_{r_{1}^{*}r_{1}^{*}}\right)+\frac{\partial \tau_{z_{1}^{*}r_{1}^{*}}}{\partial z_{1}^{*}}-\frac{\tau_{\tilde{\theta }\tilde{\theta }}}{r_{1}^{*}},$$

$z_{1}^{*}$-direction:

(4) $$\rho_{nf}\left[\frac{Dw_{1}^{*}}{Dt}\right]=-\frac{\partial p_{1}^{*}}{\partial z_{1}^{*}}+\frac{1}{r_{1}^{*}}\frac{\partial }{\partial r_{1}^{*}}\left(r_{1}^{*}\tau_{r_{1}^{*}z_{1}^{*}}\right)+\frac{\partial \tau_{z_{1}^{*}z_{1}^{*}}}{\partial z_{1}^{*}}+{\left(\rho \gamma \right)}_{nf}g\left({\tilde{T}}^{*}-{\tilde{T}}_{1}^{*}\right)-\sigma_{nf}\left(B_{0}^{2}w_{1}^{*}-E_{0}B_{0}\right),$$

**Energy Equation:**

(5) $$\begin{array}{c} {\left(\rho C_{p}\right)}_{nf}\left[\frac{\partial {\tilde{T}}^{*}}{\partial t_{1}^{*}}+u_{1}^{*}\frac{\partial {\tilde{T}}^{*}}{\partial r_{1}^{*}}+w_{1}^{*}\frac{\partial {\tilde{T}}^{*}}{\partial z_{1}^{*}}\right]=k_{eff}\left[\frac{1}{r_{1}^{*}}\frac{\partial }{\partial r_{1}^{*}}\left(r_{1}^{*}\frac{\partial {\tilde{T}}^{*}}{\partial r_{1}^{*}}\right)+\frac{\partial^{2}{\tilde{T}}^{*}}{\partial z_{1}^{*2}}\right]-\frac{\partial q_{r}^{*}}{\partial r_{1}^{*}}\\ +\sigma_{nf}{\left(B_{0}w_{1}^{*}-E_{0}B_{0}\right)}^{2}+\phi^{*}. \end{array}$$

The Casson fluid model’s rheological equation of state for an incompressible flow is as follows ([26]):

(6) $$\tau_{ij}^{*}=\left\{\begin{array}{c} 2\left(\mu_{b}^{*}+\frac{p_{y}^{*}}{\sqrt{2\pi^{*}}}\right)e_{ij}^{*},\;\;\;\;\;\;\;\;\;\;\;\;\;\pi^{*}>\pi_{c}^{*}\\ 2\left(\mu_{b}^{*}+\frac{p_{y}^{*}}{\sqrt{2\pi_{c}^{*}}}\right)e_{ij}^{*},\;\;\;\;\;\;\;\;\;\;\;\;\;\pi^{*}\leq \pi_{c}^{*} \end{array}\right.$$

where $\pi^{*}=e_{ij}^{*}.e_{ij}^{*}$ is the product of deformation rate with itself, $\pi_{c}^{*}$ is a critical value based on the non-Newtonian model, $\mu_{b}^{*}$ is the plastic dynamic viscosity of the non-Newtonian fluid, and $p_{y}^{*}$ is the yield stress of the fluid.

When $\pi^{*}\leq \pi_{c}^{*}$, Equation (6) can be expressed as:

(7) $$\tau_{ij}^{*}=2\mu_{b}^{*}\left(1+\frac{1}{\beta }\right)e_{ij}^{*}$$

where $\beta =\frac{\mu_{b}^{*}\sqrt{2\pi_{c}^{*}}}{p_{y}^{*}}$ is the Casson fluid parameter.

On incorporating the Casson fluid properties mentioned in Equation (7), the governing Equations (2)–(5) become:

**Continuity Equation:**

(8) $$\frac{\partial u_{1}^{*}}{\partial r_{1}^{*}}+\frac{u_{1}^{*}}{r_{1}^{*}}+\frac{\partial w_{1}^{*}}{\partial z_{1}^{*}}=0,$$

**Momentum Equation:**

$r_{1}^{*}$-direction:

(9) $$\begin{array}{c} \rho_{nf}\left[\frac{\partial u_{1}^{*}}{\partial t_{1}^{*}}+u_{1}^{*}\frac{\partial u_{1}^{*}}{\partial r_{1}^{*}}+w_{1}^{*}\frac{\partial u_{1}^{*}}{\partial z_{1}^{*}}\right]=-\frac{\partial p_{1}^{*}}{\partial r_{1}^{*}}+\frac{1}{r_{1}^{*}}\frac{\partial }{\partial r_{1}^{*}}\left[\mu_{eff}\left(1+\frac{1}{\beta }\right)r_{1}^{*}\frac{\partial u_{1}^{*}}{\partial r_{1}^{*}}\right]\\ +\frac{1}{2}\frac{\partial }{\partial z_{1}^{*}}\left[\mu_{eff}\left(1+\frac{1}{\beta }\right)\left(\frac{\partial w_{1}^{*}}{\partial r_{1}^{*}}+\frac{\partial u_{1}^{*}}{\partial z_{1}^{*}}\right)\right]-\mu_{eff}\left(1+\frac{1}{\beta }\right)\frac{u_{1}^{*}}{r_{1}^{*2}}, \end{array}$$

$z_{1}^{*}$-direction:

(10) $$\begin{array}{c} \rho_{nf}\left[\frac{\partial w_{1}^{*}}{\partial t_{1}^{*}}+u_{1}^{*}\frac{\partial w_{1}^{*}}{\partial r_{1}^{*}}+w_{1}^{*}\frac{\partial w_{1}^{*}}{\partial z_{1}^{*}}\right]=-\frac{\partial p_{1}^{*}}{\partial z_{1}^{*}}+\frac{1}{2}\frac{1}{r_{1}^{*}}\frac{\partial }{\partial r_{1}^{*}}\left[\mu_{eff}\left(1+\frac{1}{\beta }\right)r_{1}^{*}\left(\frac{\partial u_{1}^{*}}{\partial z_{1}^{*}}+\frac{\partial w_{1}^{*}}{\partial r_{1}^{*}}\right)\right]\\ +\frac{\partial }{\partial z_{1}^{*}}\left[\mu_{eff}\left(1+\frac{1}{\beta }\right)\frac{\partial w_{1}^{*}}{\partial z_{1}^{*}}\right]+{\left(\rho \gamma \right)}_{nf}g\left({\tilde{T}}^{*}-{\tilde{T}}_{1}^{*}\right)-\sigma_{nf}\left(B_{0}^{2}w_{1}^{*}-E_{0}B_{0}\right), \end{array}$$

**Energy Equation:**

(11) $$\begin{array}{c} {\left(\rho C_{p}\right)}_{nf}\left[\frac{\partial {\tilde{T}}^{*}}{\partial t_{1}^{*}}+u_{1}^{*}\frac{\partial {\tilde{T}}^{*}}{\partial r_{1}^{*}}+w_{1}^{*}\frac{\partial {\tilde{T}}^{*}}{\partial z_{1}^{*}}\right]=k_{eff}\left[\frac{1}{r_{1}^{*}}\frac{\partial }{\partial r_{1}^{*}}\left(r_{1}^{*}\frac{\partial {\tilde{T}}^{*}}{\partial r_{1}^{*}}\right)+\frac{\partial^{2}{\tilde{T}}^{*}}{\partial z_{1}^{*2}}\right]-\frac{\partial q_{r}^{*}}{\partial r_{1}^{*}}\\ +\sigma_{nf}{\left(B_{0}w_{1}^{*}-E_{0}B_{0}\right)}^{2}+\phi^{*},\; \end{array}$$

where

(12) $$q_{r}^{*}=-\frac{4\sigma_{e}}{3k_{e}}\frac{\partial {\tilde{T}}^{4}}{\partial r_{1}^{*}},$$

and

(13) $$\phi^{*}=2\mu_{eff}\left(1+\frac{1}{\beta }\right)\left[{\left(\frac{\partial u_{1}^{*}}{\partial r_{1}^{*}}\right)}^{2}+{\left(\frac{u_{1}^{*}}{r_{1}^{*}}\right)}^{2}+{\left(\frac{\partial w_{1}^{*}}{\partial z_{1}^{*}}\right)}^{2}+\frac{1}{2}{\left(\frac{\partial u_{1}^{*}}{\partial z_{1}^{*}}+\frac{\partial w_{1}^{*}}{\partial r_{1}^{*}}\right)}^{2}\right].$$

It is assumed that there are minimal temperature changes within the blood flow. Therefore, ${\tilde{T}}^{4}$ in Equation (12) is linearized by disregarding higher-order terms and is expanded using Taylor series around ${\tilde{T}}_{1}^{*}$:

$${\tilde{T}}^{*4}=4{\tilde{T}}_{1}^{*3}\tilde{T}-3{\tilde{T}}_{1}^{*4},$$

Hence, Equation (12) becomes

$$q_{r}^{*}=-\frac{16{\tilde{T}}_{1}^{*3}\sigma_{e}}{3k_{e}k_{f}}\frac{\partial \tilde{T}}{\partial r_{1}^{*}}.$$

The boundary conditions are ([31,48]):

(14) $$\frac{\partial w_{1}^{*}}{\partial r_{1}^{*}}=0,\frac{\partial {\tilde{T}}^{*}}{\partial r_{1}^{*}}=0\;\;\mathrm{at}\;\;r_{1}^{*}=0,$$

(15) $$w_{1}^{*}=w_{s},\;\;\frac{\partial w_{1}^{*}}{\partial r_{1}^{*}}=\frac{\alpha }{\sqrt{k_{1}^{*}}}\left(w_{s}-w_{porous}\right),\;\;{\tilde{T}}^{*}={\tilde{T}}_{w}^{*}\;\;\mathrm{at}\;\;r_{1}^{*}=R,$$

where $w_{porous}$ is the velocity in the permeable boundary, $k_{1}^{*}$ is the permeability of the medium, α is the slip parameter that is dimensionless and characterizes the structure of the permeable material within the boundary region depending on the material parameters.

The initial conditions of the flow are assumed as:

(16) $$w_{1}^{*}{{|}_{t_{1}^{*}=0}=0,{\tilde{T}}^{*}|}_{t_{1}^{*}=0}=0.$$

### 2.3. Koo-Kleinstreuer-Li (KKL) Correlation for Nanofluid Simulation

The fluid’s thermal conductivity is strongly influenced by Brownian motion. In order to account for effective thermal conductivity, Koo and Kleinstreuer [38] assumed that it consists of two parts: the general static part given by Maxwell correlation and the other is the Brownian motion part. The implications of particle size, particle volume fraction, temperature dependency, and combinations of base fluid and particles are all taken into account by this thermal conductivity model.

(17) $$k_{eff}=k_{st}+k_{Br},$$

(18) $$\frac{k_{st}}{k_{f}}=1+\frac{3\left(\frac{k_{p}}{k_{f}}-1\right)\phi }{\left(\frac{k_{p}}{k_{f}}+2\right)-\left(\frac{k_{p}}{k_{f}}-1\right)\phi },$$

Koo [39] inserted two empirical functions ($\beta_{1}$ and $\tilde{f}$) to incorporate the interaction among nanoparticles and the temperature influence in the model, resulting in:

(19) $$k_{Br}=5\times 10^{4}\beta_{1}\phi {\left(\rho C_{p}\right)}_{f}\sqrt{\frac{\kappa_{b}T}{\rho_{p}d_{p}}}\tilde{f}\left(\phi ,T\right).$$

The significance of the interfacial thermal resistance acting among base fluids and nanoparticles has been increasingly emphasized in recent years. The thin layer acting as a barrier is crucial in reducing the effective thermal conductivity of the nanoparticle, and it is thought that the thermal interfacial resistance (also known as Kapitza resistance) exists in the neighboring layers of the two distinct materials.

Li [40] reexamined the approach used by Koo and Kleinstreuer [49] and integrated the function $\tilde{f}$ and $\beta_{1}$ to create a new function $\tilde{F}$ that accounts for the particle volume fraction, diameter, and temperature. The empirical function $\tilde{F}$ depends on the type of nanofluid [40]. Additionally, a new $k_{p,eff}$ has been introduced and therefore $k_{p}$ in Equation (18) is replaced by $k_{p,eff}$ which is given by:

(20) $$R_{f}+\frac{d_{p}}{k_{p}}=\frac{d_{p}}{k_{p,eff}},$$

where $R_{f}$ is interfacial thermal resistance and has value $4\times 10^{-8}$ km^2^/W.

For CuO-Blood and Al${}_{2}$O${}_{3}$-Blood nanofluids, $\tilde{F}$ is defined as follows ([43]):

(21) $$\begin{array}{c} \tilde{F}\left(\phi ,T,d_{p}\right)=\left(b_{1}+b_{2}ln\left(d_{p}\right)+b_{3}ln\left(\phi \right)+b_{4}ln\left(\phi \right)ln\left(d_{p}\right)+b_{5}ln{\left(d_{p}\right)}^{2}\right)ln\left(T\right).\\ \;\;\;\;\;\;\;\;\;\;\;\;\;\;\;\;\;\;\;\;\;\;\;\;\;\;\;\;\;\;\;\;\;\;\;\;\;\;\;\;\;\;\;\;\;\;\;\;\;\;\;\;\;\;\;\;+(b_{6}+b_{7}ln\left(d_{p}\right)+b_{8}ln\left(\phi \right)+b_{9}ln\left(\phi \right)ln\left(d_{p}\right)+b_{10}ln{\left(d_{p}\right)}^{2}),\\ \phi \leq 0.04,300K\leq T\leq 325K \end{array}$$

with the coefficients $b_{i}$ (i=1…10) are based on the type of nanoparticles used in the analysis.

The Koo-Kleinstreuer-Li (KKL) correlation is finally written as ([49]):

(22) $$k_{Br}=5\times 10^{4}\phi \tilde{F}\left(\phi ,T,d_{p}\right){\left(\rho C_{p}\right)}_{f}\sqrt{\frac{\kappa_{b}T}{\rho_{p}d_{p}}}.$$

The effective viscosity of the nanofluid is given as ([49]):

(23) $$\mu_{eff}=\mu_{st}+\mu_{Br}=\mu_{st}+\frac{k_{Br}}{k_{f}}\times \frac{\mu_{f}}{Pr},$$

where,

$$\mu_{st}=\frac{\mu_{f}}{{\left(1-\phi_{1}\right)}^{2.5}}.$$

Here, the Reynold’s viscosity model [50] is considered to address the fluid’s viscosity $\mu_{f}$ given by:

(24) $$\mu_{f}\left(\tilde{\theta }\right)=\mu_{0}e^{-\beta_{0}\tilde{\theta }}=\mu_{0}\left[1-\beta_{0}\tilde{\theta }\right]\;\;\mathrm{where}\;\;\beta_{0}<<1.$$

Therefore,

$$\mu_{eff}=\frac{\mu_{0}\left(1-\beta_{0}\tilde{\theta }\right)}{{\left(1-\phi_{1}\right)}^{2.5}}+\frac{k_{Br}}{k_{f}}\times \frac{\mu_{0}\left(1-\beta_{0}\tilde{\theta }\right)}{Pr}$$

(25) $$\mu_{eff}=\mu_{0}\left(1-\beta_{0}\tilde{\theta }\right)\left(\frac{1}{{\left(1-\phi_{1}\right)}^{2.5}}+\frac{k_{Br}}{k_{f}Pr}\right).$$

**Thermophysical Features of Nanofluid**

The density ($\rho_{nf}$), heat capacity (${\left(\rho C_{p}\right)}_{nf}$), electrical conductivity ($\sigma_{nf}$) and thermal expansion coefficient ($\gamma_{nf}$) of nanofluid are given by the usual relations as follows ([34,51]):

(26) $$\rho_{nf}=\left(1-\phi \right)\rho_{f}+\phi \rho_{p}$$

(27) $${\left(\rho C_{p}\right)}_{nf}=\left(1-\phi \right){\left(\rho C_{p}\right)}_{f}+\phi {\left(\rho C_{p}\right)}_{p}$$

(28) $$\sigma_{nf}=\sigma_{f}\left(1+\frac{3(\sigma -1)\phi }{(\sigma +2)-(\sigma -1)\phi }\right),\;\;\;\;\sigma =\frac{\sigma_{p}}{\sigma_{f}}.$$

(29) $$\gamma_{nf}=\left(1-\phi \right){\left(\gamma \right)}_{f}+\phi_{1}{\left(\gamma \right)}_{p}$$

### 2.4. Nanoparticle and Base Fluid Features

Table 1 lists the thermophysical characteristics of the blood and nanoparticles utilized in the study, and Table 2 provides the coefficient values of copper oxide and aluminum oxide.

### 2.5. Non-Dimensional Analysis

The governing equations given by (8)–(11) need to be transformed into dimensionless form so that a numerical solution is obtained. The non-dimensional variables are introduced as:

(30) $$\begin{array}{c} {\bar{r}}_{1}^{*}=\frac{r_{1}^{*}}{R_{0}},{\bar{w}}_{1}^{*}=\frac{w_{1}^{*}}{U_{0}},{\bar{u}}_{1}^{*}=\frac{L_{0}u_{1}^{*}}{\delta^{*}U_{0}},{\bar{t}}_{1}^{*}=\frac{U_{0}t_{1}^{*}}{R_{0}},{\bar{z}}_{1}^{*}=\frac{z_{1}^{*}}{L_{0}},{\bar{p}}_{1}^{*}=\frac{R_{0}^{2}p_{1}^{*}}{U_{0}L_{0}\mu_{f}},\tilde{\theta }=\frac{{\tilde{T}}^{*}-{\tilde{T}}_{1}^{*}}{{\tilde{T}}_{w}^{*}-{\tilde{T}}_{1}^{*}},\bar{R}=\frac{R}{R_{0}},\\ \alpha =\frac{\alpha^{*}L_{0}}{R_{0}},\bar{d}=\frac{d}{L_{0}},{\bar{w}}_{s}=\frac{w_{s}}{U_{0}},Re=\frac{U_{0}\rho_{f}R_{0}}{\mu_{f}},M^{2}=\frac{\sigma_{f}B_{0}^{2}R_{0}^{2}}{\mu_{f}},Gr=\frac{\rho_{f}R_{0}^{2}g\gamma_{f}\left({\tilde{T}}_{w}^{*}-{\tilde{T}}_{1}^{*}\right)}{\mu_{f}U_{0}},\\ Ec=\frac{U_{0}^{2}}{C_{p}\left({\tilde{T}}_{w}^{*}-{\tilde{T}}_{1}^{*}\right)},Pr=\frac{\mu_{f}C_{p}}{k_{f}},Nr=\frac{16\sigma_{e}{\tilde{T}}_{1}^{*3}}{3k_{f}k_{e}}. \end{array}$$

The insertion of the above non-dimensional parameters mentioned in (18), disregarding the bars and using the mild stenotic hypotheses that the maximal stenosis height is less than the artery’s radius, i.e., $\delta (=\delta^{*}/R_{0})<<1$, and the artery’s radius and the stenotic region’s length are proportionate, i.e., $\epsilon \left(=R_{0}/L_{0}\right)=O\left(1\right)$ leads to the modified form of governing Equations (8)–(11), which are as follows:

**Continuity Equation:**

(31) $$\frac{\partial w_{1}^{*}}{\partial z_{1}^{*}}=0,$$

**Momentum Equation:**

**r-direction:**

(32) $$\frac{\partial p_{1}^{*}}{\partial r_{1}^{*}}=0,$$

**z-direction:**

(33) $$Re\frac{\rho_{nf}}{\rho_{f}}\frac{\partial w_{1}^{*}}{\partial t_{1}^{*}}=-\frac{\partial p_{1}^{*}}{\partial z_{1}^{*}}+\frac{1}{2r_{1}^{*}}\frac{\partial }{\partial r_{1}^{*}}\left[\frac{\mu_{eff}}{\mu_{0}}\left(1+\frac{1}{\beta }\right)r_{1}^{*}\frac{\partial w_{1}^{*}}{\partial r_{1}^{*}}\right]+\frac{{\left(\rho \gamma \right)}_{nf}}{{\left(\rho \gamma \right)}_{f}}Gr\tilde{\theta }-\frac{\sigma_{nf}}{\sigma_{f}}M^{2}\left(w_{1}^{*}-E_{1}^{*}\right),$$

**Energy Equation:**

(34) $$\begin{array}{c} \frac{{\left(\rho C_{p}\right)}_{nf}}{{\left(\rho C_{p}\right)}_{f}}\frac{\partial \tilde{\theta }}{\partial t_{1}^{*}}=\frac{1}{RePr}\frac{k_{eff}}{k_{f}}\left[\frac{\partial^{2}\tilde{\theta }}{\partial r_{1}^{*2}}+\frac{1}{r_{1}^{*}}\frac{\partial \tilde{\theta }}{\partial r_{1}^{*}}\right]+\frac{Nr}{RePr}\frac{\partial^{2}\tilde{\theta }}{\partial r_{1}^{*2}}+\frac{\sigma_{nf}}{\sigma_{f}}\frac{EcM^{2}}{Re}{\left(w_{1}^{*}-E_{1}^{*}\right)}^{2}\\ +\frac{\mu_{eff}}{\mu_{0}}\left(1+\frac{1}{\beta }\right)\frac{Ec}{Re}\left[{\left(\frac{\partial w_{1}^{*}}{\partial r_{1}^{*}}\right)}^{2}\right]. \end{array}$$

The geometry of stenosis, aneurysm and boundary conditions in the dimensionless form can be described as ([48]):

(35) $$R\left(z_{1}^{*}\right)=\left\{\begin{array}{c} \left[1-2\delta^{*}\left[cos\left(2\pi \left(\frac{z_{1}^{*}-d}{2}-\frac{1}{4}\right)\right)-\frac{7}{100}cos(32\pi (z_{1}^{*}-d-\frac{1}{2}))\right]\right],\;\;\;\;\;d\leq z_{1}^{*}\leq d+1,\\ \left[1+2\delta^{*}\left[cos\left(2\pi \left(\frac{z_{1}^{*}-d}{2}-\frac{1}{4}\right)\right)-\frac{7}{100}cos(32\pi (z_{1}^{*}-d-\frac{1}{2}))\right]\right],\;\;d+2\leq z_{1}^{*}\leq d+3,\\ 1,\;\;\;\;\;\;\;\;\;\;\;\;\;\;\;\;\;\;\;\;\;\;\;\;\;\;\;\;\;\;\;\;\;\;\;\;\;\;\;\;\;\;\;\;\;\;\;\;\;\;\;\;\;\;\;\;\;\;\;\;\;\;\;\mathrm{otherwise}. \end{array}\right.$$

(36) $$\frac{\partial w_{1}^{*}}{\partial r_{1}^{*}}{|}_{r_{1}^{*}=0}=0,w_{1}^{*}\left(r_{1}^{*},t_{1}^{*}\right){|}_{r_{1}^{*}=R}=w_{s},\frac{\partial w_{1}^{*}}{\partial r_{1}^{*}}{|}_{r_{1}^{*}=R}=\frac{\alpha }{\sqrt{Da}}\left(w_{s}-w_{porous}\right),$$

where

$$w_{porous}=-\frac{Da}{\mu_{nf}}\frac{dp}{dz}.$$

Blood flows through the cardiovascular system due to the heart’s pumping motion, causing a pressure gradient across the vascular network. The pressure gradient is separated into two parts: non-fluctuating (continuous) and fluctuating (pulsatile) [52] as given below:

(37) $$-\frac{\partial p_{1}^{*}}{\partial z_{1}^{*}}=A_{0}+A_{1}cos\left(w_{p}t_{1}^{*}\right),t_{1}^{*}>0,$$

where, $A_{0}$ and $A_{1}$ signify the amplitudes of the steady-state and pulsatile pressure gradient components, respectively, and $w_{p}=2\pi f_{p}$, $f_{p}$ depicts the heart pulse frequency.

On the substitution of dimensionless variables in (37), the modified equation for the pressure gradient becomes:

(38) $$-\frac{\partial p_{1}^{*}}{\partial z_{1}^{*}}=B_{1}\left[1+ecos\left(c_{1}t_{1}^{*}\right)\right],$$

where

(39) $$e=\frac{A_{1}}{A_{0}},B_{1}=\frac{A_{0}R_{0}^{2}}{\mu_{0}U_{0}},c_{1}=\frac{2\pi R_{0}f_{p}}{U_{0}}.$$

Using Equation (38) in Equation (33), we have:

(40) $$\begin{array}{c} Re\frac{\rho_{nf}}{\rho_{f}}\frac{\partial w_{1}^{*}}{\partial t_{1}^{*}}=B_{1}\left[1+ecos\left(c_{1}t_{1}^{*}\right)\right]+\frac{1}{2r_{1}^{*}}\left(1+\frac{1}{\beta }\right)\left(\frac{1}{{\left(1-\phi_{1}\right)}^{2.5}}+\frac{k_{Br}}{k_{f}Pr}\right)\frac{\partial }{\partial r_{1}^{*}}\left[\left(1-\beta_{0}\tilde{\theta }\right)r_{1}^{*}\frac{\partial w_{1}^{*}}{\partial r_{1}^{*}}\right]\\ +\frac{{\left(\rho \gamma \right)}_{nf}}{{\left(\rho \gamma \right)}_{f}}Gr\tilde{\theta }-\frac{\sigma_{nf}}{\sigma_{f}}M^{2}\left(w_{1}^{*}-E_{1}^{*}\right). \end{array}$$

### 2.6. Radial Coordinate Transformation

The physical geometry taken into account in the formulated problem is cylindrical, i.e., a cylindrical coordinate system is considered. However, in order to use the computational approach, the considered geometry needs to be transformed into a rectangular domain by employing the transformation $\left(x_{1}^{*}=\frac{r_{1}^{*}}{R(z_{1}^{*})}\right)$. On applying this transformation, the Equations (40) and (34) become:

(41) $$\begin{array}{c} Re\frac{\rho_{nf}}{\rho_{f}}\frac{\partial w_{1}^{*}}{\partial t_{1}^{*}}=B_{1}\left[1+ecos\left(c_{1}t_{1}^{*}\right)\right]+\frac{1}{2R^{2}}\left(1+\frac{1}{\beta }\right)\left(\frac{1}{{\left(1-\phi_{1}\right)}^{2.5}}+\frac{k_{Br}}{k_{f}Pr}\right)\\ \times \left[\left(1-\beta_{0}\tilde{\theta }\right)\left(\frac{\partial^{2}w_{1}^{*}}{\partial x_{1}^{*2}}+\frac{1}{x_{1}^{*}}\frac{\partial w_{1}^{*}}{\partial x_{1}^{*}}\right)-\beta_{0}\frac{\partial \tilde{\theta }}{\partial x_{1}^{*}}\frac{\partial w_{1}^{*}}{\partial x_{1}^{*}}\right]+\frac{{\left(\rho \gamma \right)}_{nf}}{{\left(\rho \gamma \right)}_{f}}Gr\tilde{\theta }-\frac{\sigma_{nf}}{\sigma_{f}}M^{2}\left(w_{1}^{*}-E_{1}^{*}\right), \end{array}$$

(42) $$\begin{array}{c} \frac{{\left(\rho C_{p}\right)}_{nf}}{{\left(\rho C_{p}\right)}_{f}}\frac{\partial \tilde{\theta }}{\partial t_{1}^{*}}=\frac{1}{RePr}\frac{k_{eff}}{k_{f}}\left(\frac{1}{R^{2}}\right)\left[\frac{\partial^{2}\tilde{\theta }}{\partial x_{1}^{*2}}+\frac{1}{x_{1}^{*}}\frac{\partial \tilde{\theta }}{\partial x_{1}^{*}}\right]+\left(\frac{1}{R^{2}}\right)\frac{Nr}{RePr}\frac{\partial^{2}\tilde{\theta }}{\partial x_{1}^{*2}}+\frac{\sigma_{nf}}{\sigma_{f}}\frac{EcM^{2}}{Re}{\left(w_{1}^{*}-E_{1}^{*}\right)}^{2}\\ +\frac{1}{R^{2}}\left(1+\frac{1}{\beta }\right)\left(\frac{Ec}{Re}\right)\left(1-\beta_{0}\tilde{\theta }\right)\left(\frac{1}{{\left(1-\phi_{1}\right)}^{2.5}}+\frac{k_{Br}}{k_{f}Pr}\right){\left(\frac{\partial w_{1}^{*}}{\partial x_{1}^{*}}\right)}^{2}. \end{array}$$

The wall shear stress (WSS), volumetric flow rate, and resistance impedance are expressed as:

(43) $$\tau_{w}=-\frac{1}{R}{\left(\frac{\partial w_{1}^{*}}{\partial x_{1}^{*}}\right)}_{x_{1}^{*}=1},$$

(44) $$Q_{1}=2\pi R^{2}\int_{0}^{1}w_{1}^{*}x_{1}^{*}dx_{1}^{*},$$

(45) $$\lambda =\frac{L\left(\frac{\partial p_{1}^{*}}{\partial z_{1}^{*}}\right)}{Q_{1}}.$$

## 3. Numerical Procedure

The partial differential Equations (41) and (42) are coupled differential equations, therefore obtaining an analytic solution is too difficult. On the other hand, numerical approaches can yield a highly accurate solution. An unconditionally stable implicit finite difference (Crank–Nicolson) approach is used in this case. The superscripts and subscripts are not taken into account throughout the discretization process for Equations (41) and (42).

### 3.1. Discretization

On employing the values of thermophysical features of nanofluid and discretizing the governing Equations (41) and (42) using Crank-Nicolson scheme, the desired form of equations is:

(46) $$\begin{array}{c} \left[\left(1-\phi \right)+\phi \frac{\rho_{p}}{\rho_{f}}\right]Re\left[\frac{w_{i}^{k+1}-w_{i}^{k}}{dt}\right]=B_{1}\left[1+ecos\left(c_{1}t^{k}\right)\right]+\frac{1}{2}\left(1+\frac{1}{\beta }\right)\left(\frac{1}{R^{2}}\right)\left(1-\beta_{0}{\tilde{\theta }}_{i}^{k}\right)\\ \times \left(\frac{1}{{\left(1-\phi_{1}\right)}^{2.5}}+\frac{k_{Brownian}}{k_{f}Pr}\right)\times [\frac{1}{2}\left(\frac{w_{i+1}^{k+1}-2w_{i}^{k+1}+w_{i-1}^{k+1}}{dx^{2}}+\frac{w_{i+1}^{k}-2w_{i}^{k}+w_{i-1}^{k}}{dx^{2}}\right)\\ +\frac{1}{2x(i)}\left(\frac{w_{i+1}^{k+1}-w_{i-1}^{k+1}}{2dx}+\frac{w_{i+1}^{k}-w_{i-1}^{k}}{2dx}\right)]-\frac{\beta_{0}}{2R^{2}}\left(1+\frac{1}{\beta }\right)\left(\frac{1}{{\left(1-\phi_{1}\right)}^{2.5}}+\frac{k_{Br}}{k_{f}Pr}\right)\\ \times \left[\left(\frac{{\tilde{\theta }}_{i+1}^{k+1}-{\tilde{\theta }}_{i-1}^{k+1}}{4dx}+\frac{{\tilde{\theta }}_{i+1}^{k}-{\tilde{\theta }}_{i-1}^{k}}{4dx}\right)\left(\frac{w_{i+1}^{k+1}-w_{i-1}^{k+1}}{4dx}+\frac{w_{i+1}^{k}-w_{i-1}^{k}}{4dx}\right)\right]\\ +\left[\left(1-\phi \right)+\phi \frac{{\left(\rho \gamma \right)}_{p}}{{\left(\rho \gamma \right)}_{f}}\right]Gr{\tilde{\theta }}_{i}^{k}-\frac{1}{2}\frac{\sigma_{nf}}{\sigma_{f}}M^{2}\left(w_{i}^{k}+w_{i}^{k+1}\right)+\frac{\sigma_{nf}}{\sigma_{f}}M^{2}E_{1}^{*}, \end{array}$$

(47) $$\begin{array}{c} \left[\left(1-\phi \right)+\phi \frac{{\left(\rho C_{p}\right)}_{p}}{{\left(\rho C_{p}\right)}_{f}}\right]\left[\frac{{\tilde{\theta }}_{i}^{k+1}-{\tilde{\theta }}_{i}^{k}}{dt}\right]=\frac{1}{RePr}\frac{k_{nf}}{k_{f}}\left(\frac{1}{R^{2}}\right)[\frac{1}{2}(\frac{{\tilde{\theta }}_{i+1}^{k+1}-2{\tilde{\theta }}_{i}^{k+1}+{\tilde{\theta }}_{i-1}^{k+1}}{dx^{2}}\\ +\frac{{\tilde{\theta }}_{i+1}^{k}-2{\tilde{\theta }}_{i}^{k}+{\tilde{\theta }}_{i-1}^{k}}{dx^{2}})+\frac{1}{2x(i)}\left(\frac{{\tilde{\theta }}_{i+1}^{k+1}-{\tilde{\theta }}_{i-1}^{k+1}}{2dx}+\frac{{\tilde{\theta }}_{i+1}^{k}-{\tilde{\theta }}_{i-1}^{k}}{2dx}\right)]\\ +\left(\frac{1}{2R^{2}}\right)\frac{Nr}{RePr}\left(\frac{{\tilde{\theta }}_{i+1}^{k+1}-2{\tilde{\theta }}_{i}^{k+1}+{\tilde{\theta }}_{i-1}^{k+1}}{dx^{2}}+\frac{{\tilde{\theta }}_{i+1}^{k}-2{\tilde{\theta }}_{i}^{k}+{\tilde{\theta }}_{i-1}^{k}}{dx^{2}}\right)+\left(1+\frac{1}{\beta }\right)\left(\frac{1}{R^{2}}\right)\\ \times \left(1-\frac{\beta_{0}}{2}\left({\tilde{\theta }}_{i}^{k+1}+{\tilde{\theta }}_{i}^{k}\right)\right)\left(\frac{1}{{\left(1-\phi_{1}\right)}^{2.5}}+\frac{k_{Br}}{k_{f}Pr}\right){\left[\frac{1}{2}\left(\frac{w_{i+1}^{k+1}-w_{i-1}^{k+1}}{2dx}+\frac{w_{i+1}^{k}-w_{i-1}^{k}}{2dx}\right)\right]}^{2}\\ +\frac{\sigma_{nf}}{\sigma_{f}}\frac{EcM^{2}}{Re}{\left[\frac{1}{2}(w_{i}^{k+1}+w_{i}^{k})-E_{1}^{*}\right]}^{2}. \end{array}$$

The Crank-Nicolson scheme employed in the current analysis is, however, stable for all values for dt and dx still, a minimal value is considered with great precision as $dt=10^{-4}$ and $dx=10^{-4}$. It is noticed that no further change occurs in the values of hemodynamical parameters studied in the research with decreasing values of dt and dx. A total of N+1 grid points have been considered in the spatial direction, with x=1/(N+1) being the step size, whereas M+1 grid points are considered temporal. The value at any time instant $t^{k}$ is given as $t^{k}=\left(k-1\right)dt$, dt being a small increment in time. As the scheme employed is an implicit one; therefore a system of equations is obtained, and it is in the form of a tri-diagonal system which can be solved with the Tri-diagonal Matrix Algorithm (TDMA) [53].

Equation (46) corresponds to a tri-diagonal system, which is given by

(48) $$A_{i}^{k}w_{i-1}^{k+1}+B_{i}^{k}w_{i}^{k+1}+C_{i}^{k}w_{i+1}^{k+1}=A_{i}^{{}^{\prime }k}w_{i-1}^{k}+B_{i}^{{}^{\prime }k}w_{i}^{k}+C_{i}^{{}^{\prime }k}w_{i+1}^{k}+D_{i}^{k},$$

where

$A_{i}^{k}=-(1+\frac{1}{\beta })\left(\frac{\left(1-\beta_{0}{\tilde{\theta }}_{i}^{k}\right)}{{\left(1-\phi \right)}^{2.5}}+\frac{k_{Br}}{k_{f}Pr}\right)(\frac{1}{4R^{2}})(\frac{dt}{dx^{2}}-\frac{1}{2x(i)}\frac{dt}{dx})-\frac{\beta_{0}}{8R^{2}}(\frac{dt}{dx})(1+\frac{1}{\beta })(\frac{1}{{\left(1-\phi_{1}\right)}^{2.5}}+\frac{k_{Br}}{k_{f}Pr})\times (\frac{{\tilde{\theta }}_{i+1}^{k+1}-{\tilde{\theta }}_{i-1}^{k+1}}{2dx}+\frac{{\tilde{\theta }}_{i+1}^{k}-{\tilde{\theta }}_{i-1}^{k}}{2dx})$, 

$B_{i}^{k}=Re\left[\left(1-\phi \right)+\phi \frac{\rho_{p}}{\rho_{f}}\right]+(\frac{1}{2R^{2}})(\frac{dt}{dx^{2}})(1+\frac{1}{\beta })(\frac{\left(1-\beta_{0}{\tilde{\theta }}_{i}^{k}\right)}{{\left(1-\phi \right)}^{2.5}}+\frac{k_{Br}}{k_{f}Pr})+\frac{dt}{2}\frac{\sigma_{nf}}{\sigma_{f}}M^{2}$, 

$C_{i}^{k}=-(1+\frac{1}{\beta })\left(\frac{\left(1-\beta_{0}{\tilde{\theta }}_{i}^{k}\right)}{{\left(1-\phi \right)}^{2.5}}+\frac{k_{Br}}{k_{f}Pr}\right)(\frac{1}{4R^{2}})(\frac{dt}{dx^{2}}+\frac{1}{2x(i)}\frac{dt}{dx})+\frac{\beta_{0}}{8R^{2}}(\frac{dt}{dx})(1+\frac{1}{\beta })(\frac{1}{{\left(1-\phi_{1}\right)}^{2.5}}+\frac{k_{Br}}{k_{f}Pr})\times (\frac{{\tilde{\theta }}_{i+1}^{k+1}-{\tilde{\theta }}_{i-1}^{k+1}}{2dx}+\frac{{\tilde{\theta }}_{i+1}^{k}-{\tilde{\theta }}_{i-1}^{k}}{2dx})$, 

$A_{i}^{{}^{\prime }k}=(1+\frac{1}{\beta })\left(\frac{\left(1-\beta_{0}{\tilde{\theta }}_{i}^{k}\right)}{{\left(1-\phi \right)}^{2.5}}+\frac{k_{Br}}{k_{f}Pr}\right)(\frac{1}{4R^{2}})(\frac{dt}{dx^{2}}-\frac{1}{2x(i)}\frac{dt}{dx})+\frac{\beta_{0}}{8R^{2}}(\frac{dt}{dx})(1+\frac{1}{\beta })(\frac{1}{{\left(1-\phi_{1}\right)}^{2.5}}+\frac{k_{Br}}{k_{f}Pr})\times (\frac{{\tilde{\theta }}_{i+1}^{k+1}-{\tilde{\theta }}_{i-1}^{k+1}}{2dx}+\frac{{\tilde{\theta }}_{i+1}^{k}-{\tilde{\theta }}_{i-1}^{k}}{2dx})$, 

$B_{i}^{{}^{\prime }k}=Re\left[\left(1-\phi \right)+\phi \frac{\rho_{p}}{\rho_{f}}\right]-(\frac{1}{2R^{2}})(\frac{dt}{dx^{2}})(1+\frac{1}{\beta })\left(\frac{\left(1-\beta_{0}{\tilde{\theta }}_{i}^{k}\right)}{{\left(1-\phi \right)}^{2.5}}+\frac{k_{Br}}{k_{f}Pr}\right)-\frac{dt}{2}\frac{\sigma_{nf}}{\sigma_{f}}M^{2}$, 

$C_{i}^{{}^{\prime }k}=(1+\frac{1}{\beta })\left(\frac{\left(1-\beta_{0}{\tilde{\theta }}_{i}^{k}\right)}{{\left(1-\phi \right)}^{2.5}}+\frac{k_{Br}}{k_{f}Pr}\right)(\frac{1}{4R^{2}})(\frac{dt}{dx^{2}}+\frac{1}{2x(i)}\frac{dt}{dx})-\frac{\beta_{0}}{8R^{2}}(\frac{dt}{dx})(1+\frac{1}{\beta })(\frac{1}{{\left(1-\phi_{1}\right)}^{2.5}}+\frac{k_{Br}}{k_{f}Pr})\times (\frac{{\tilde{\theta }}_{i+1}^{k+1}-{\tilde{\theta }}_{i-1}^{k+1}}{2dx}+\frac{{\tilde{\theta }}_{i+1}^{k}-{\tilde{\theta }}_{i-1}^{k}}{2dx})$, 

$D_{i}^{k}=dtB_{1}\left[1+ecos\left(c_{1}t^{k}\right)\right]+dt\left[\left(1-\phi \right)+\phi \frac{{\left(\rho \gamma \right)}_{p}}{{\left(\rho \gamma \right)}_{f}}\right]Gr{\tilde{\theta }}_{i}^{k}+dt\frac{\sigma_{nf}}{\sigma_{f}}M^{2}E_{1}^{*}$. 

The tri-diagonal system corresponding to Equation (47) is given by

(49) $$P_{i}^{k}\theta_{i-1}^{k+1}+Q_{i}^{k}\theta_{i}^{k+1}+S_{i}^{k}\theta_{i+1}^{k+1}=P_{i}^{{}^{\prime }k}\theta_{i-1}^{k}+Q_{i}^{{}^{\prime }k}\theta_{i}^{k}+S_{i}^{{}^{\prime }k}\theta_{i+1}^{k}+F_{i}^{k},$$

where

$P_{i}^{k}=-\frac{1}{RePr}\left(\frac{1}{2R^{2}}\right)\left[\frac{k_{nf}}{k_{f}}\left(\frac{dt}{dx^{2}}-\frac{1}{2x(i)}\frac{dt}{dx}\right)+Nr\frac{dt}{dx^{2}}\right]$, 

$Q_{i}^{k}=\left[\left(1-\phi \right)+\phi \frac{{\left(\rho C_{p}\right)}_{p}}{{\left(\rho C_{p}\right)}_{f}}\right]+\frac{1}{RePr}\frac{1}{R^{2}}\frac{dt}{dx^{2}}\left(\frac{k_{nf}}{k_{f}}+Nr\right)+dt\frac{\beta_{0}}{{\left(1-\phi \right)}^{2.5}}\left(1+\frac{1}{\beta }\right)\frac{Ec}{Re}\left(\frac{1}{2R^{2}}\right)\times {\left[\frac{1}{2}\left(\frac{w_{i+1}^{k+1}-w_{i-1}^{k+1}}{2dx}+\frac{w_{i+1}^{k}-w_{i-1}^{k}}{2dx}\right)\right]}^{2}$, 

$S_{i}^{k}=-\frac{1}{RePr}\left(\frac{1}{2R^{2}}\right)\left[\frac{k_{nf}}{k_{f}}\left(\frac{dt}{dx^{2}}+\frac{1}{2x(i)}\frac{dt}{dx}\right)+Nr\frac{dt}{dx^{2}}\right]$, 

$P_{i}^{{}^{\prime }k}=\frac{1}{RePr}\left(\frac{1}{2R^{2}}\right)\left[\frac{k_{nf}}{k_{f}}\left(\frac{dt}{dx^{2}}-\frac{1}{2x(i)}\frac{dt}{dx}\right)+Nr\frac{dt}{dx^{2}}\right]$, 

$Q_{i}^{{}^{\prime }k}=\left[\left(1-\phi \right)+\phi \frac{{\left(\rho C_{p}\right)}_{p}}{{\left(\rho C_{p}\right)}_{f}}\right]-\frac{1}{RePr}\frac{1}{R^{2}}\frac{dt}{dx^{2}}\left(\frac{k_{nf}}{k_{f}}+Nr\right)-dt\frac{\beta_{0}}{{\left(1-\phi \right)}^{2.5}}\left(1+\frac{1}{\beta }\right)\frac{Ec}{Re}\left(\frac{1}{2R^{2}}\right)\times {\left[\frac{1}{2}\left(\frac{w_{i+1}^{k+1}-w_{i-1}^{k+1}}{2dx}+\frac{w_{i+1}^{k}-w_{i-1}^{k}}{2dx}\right)\right]}^{2}$,

$S_{i}^{{}^{\prime }k}=\frac{1}{RePr}\left(\frac{1}{2R^{2}}\right)\left[\frac{k_{nf}}{k_{f}}\left(\frac{dt}{dx^{2}}+\frac{1}{2x(i)}\frac{dt}{dx}\right)+Nr\frac{dt}{dx^{2}}\right]$.

$F_{i}^{k}=dt\frac{\sigma_{nf}}{\sigma_{f}}\frac{EcM^{2}}{Re}{\left[\frac{1}{2}(w_{i}^{k+1}+w_{i}^{k})-E_{1}^{*}\right]}^{2}+dt(1+\frac{1}{\beta })\left(\frac{1}{{\left(1-\phi \right)}^{2.5}}+\frac{k_{Brownian}}{k_{f}Pr_{f}}\right)\frac{Ec}{Re}\left(\frac{1}{R^{2}}\right)\times {\left[\frac{1}{2}\left(\frac{w_{i+1}^{k+1}-w_{i-1}^{k+1}}{2dx}+\frac{w_{i+1}^{k}-w_{i-1}^{k}}{2dx}\right)\right]}^{2}$.

### 3.2. Validation of the Employed Numerical Scheme

The Crank-Nicolson finite difference method has been applied in the present work. To check the accuracy of the applied numerical scheme, two methods have been adopted. Firstly, a mesh independence test is performed, which includes both grid independence as well as time independence. Also, validation has been performed with the existing model of Zaman et al. [54].

#### 3.2.1. Mesh Independence

A “grid-independency test” is used to optimize the suggested grid system for the current study, allowing for the selection of a mesh density that is both computationally accurate and economically acceptable. Table 3 lists the ideal grid size (100 × 100) that achieves enough precision; any other mesh size refinement does not provide an increase in accuracy. In accordance with Table 4, the “time-independency test” provides the best time-step size of dt = 0.01.

#### 3.2.2. Validation with Existing Literature

**Figure 2:**
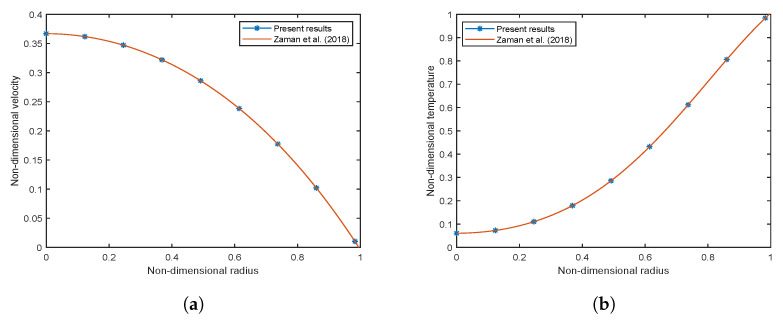
Comparison results (**a**) non-dimensional velocity for Gr = 0.5, (**b**) non-dimensional temperature for $\phi_{2}$ = 0.01.

The accuracy of our results has been verified by validating it with the existing model of Zaman et al. [54]. The effect of EMHD, Joule heating, viscous dissipation, and radiation has been neglected to verify the results with the simplest existing model of Zaman et al. [54]. As the Casson fluid parameter in the current work approaches infinity (β→∞), the current model approaches the Newtonian model in [54]. The KKL scheme adopted for variable viscosity and thermal conductivity is also replaced by a generalized model for nanofluids. The results have been compared for Al${}_{2}$O${}_{3}$ nanoparticles, which are common in both research work. For verification, Figure 2a,b have been plotted. A comparison with the existing literature [54] reveals a good agreement to support the validity of the present solutions.

## 4. Results and Graphical Analysis

In this section, the impact of various flow parameters such as electric field parameter ($E_{1}^{*}$), viscosity parameter ($\beta_{0}$), volume fractions of both the nanoparticles ($\phi_{1},\phi_{2}$), Casson fluid parameter (β), radiation parameter (Nr), Eckert number (Ec), and Prandtl number (Pr) on non-dimensional velocity, wall shear stress, flow rate, non-dimensional temperature, and Nusselt number is analyzed. A comparison of CuO-Blood and Al${}_{2}$O${}_{3}$-Blood nanofluid has been conducted. The default values of the emerging parameters are mentioned in Table 5, and the range of non-dimensional parameters considered in the present analysis are depicted in Table 6.

### 4.1. Nusselt Number and Enhancement Ratio

The ratio of thermal energy conducted in the fluid to thermal energy undertaken in the fluid is expressed by the Nusselt number. It represents the convective heat transfer taking place at the surface and is equivalent to the dimensionless temperature gradient at the surface. Therefore, the Nusselt number is mathematically represented as:

(50) $$Nu=\sqrt{Re}\frac{k_{eff}}{k_{f}}\left(1+Nr\right)\frac{1}{R}{\left(\frac{\partial \tilde{\theta }}{\partial x_{1}^{*}}\right)}_{x_{1}^{*}=1}.$$

The temperature plays a vital role during any treatment or surgery; therefore, regulating it is the primary concern of physicians. The two types of nanoparticles are considered in the present study, namely CuO and Al${}_{2}$O${}_{3}$ nanoparticles. They both have opposing effects on the temperature profile. Therefore, a relative % variation has been performed to decide which nanoparticle will be suitable in the required situation and is given by:

(51) $$\mathrm{Relative}\;\%\;\mathrm{Variation}=\frac{Nu\left(Al_{2}O_{3}\right)-Nu\left(CuO\right)}{Nu(Al_{2}O_{3})}\times 100$$

**Figure 3:**
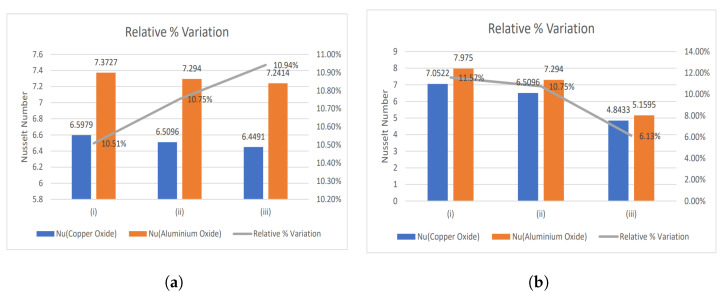
Relative % variation for (**a**) Magnetic number (i) $M^{2}$ = 1, (ii) $M^{2}$ = 3, (iii) $M^{2}$ = 5, and (**b**) Electric field parameter (i) $E_{1}^{*}$ = 0.1, (ii) $E_{1}^{*}$ = 0.3, (iii) $E_{1}^{*}$ = 0.6.

**Figure 4:**
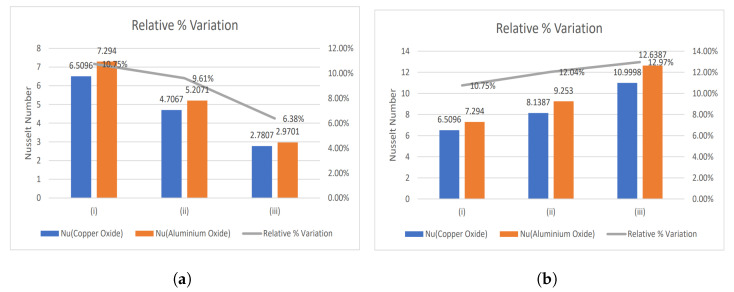
Relative % variation for (**a**) Eckert number (i) Ec = 0.2, (ii) Ec = 0.4, (iii) Ec = 0.6, and (**b**) Radiation parameter (i) Nr = 0.5, (ii) Nr = 1, (iii) Nr = 2.

**Figure 5:**
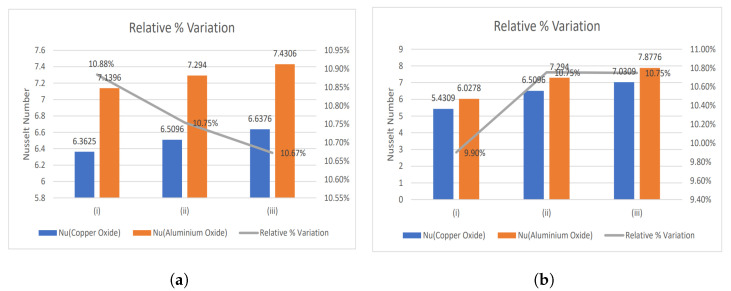
Relative % variation for (**a**) Casson fluid parameter (i) β = 1, (ii) β = 2, (iii) β = 10, and (**b**) Prandtl number (i) Pr = 14, (ii) Pr = 21, (iii) Pr = 25.

Figure 3, Figure 4 and Figure 5 show relative % error for various influential parameters, namely Magnetic number $M^{2}$, electric field parameter $E_{1}^{*}$, Eckert number Ec, radiation parameter Nr, Casson fluid parameter β, and Prandtl number Pr. Figure 3a depicts that a decreasing trend in Nusselt number is analyzed with increasing values of $M^{2}$ whereas the relative % variation show enhancement as $M^{2}$ varies from 1 to 5. The Nusselt number and relative % variation decline with increasing $E_{1}^{*}$ values as shown in Figure 3b. Figure 4 illustrates that relative % variation declines with rising Eckert number values. In contrast, an opposite trend is analyzed with proliferating radiation parameter values, i.e., relative % variation booms with mounting Nr values. The Nusselt number inclines whereas relative % variation declines with enlarging β values as indicated by Figure 5a. Figure 5b exhibits an increase in Nusselt number and relative % variation with ascending values of Prandtl number.

### 4.2. Influence of Nanoparticle Diameter and Volume Fraction

**Figure 6:**
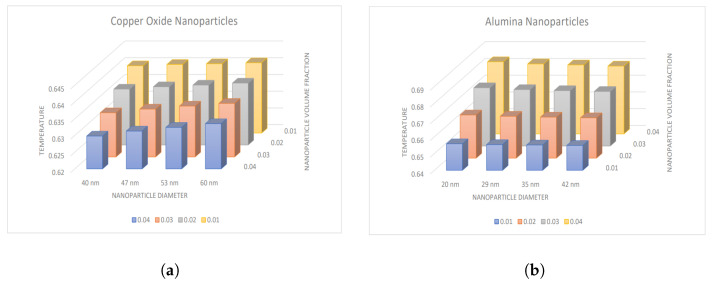
Variation in temperature with nanoparticle diameter and volume fraction for (**a**) CuO-nanoparticles, (**b**) Al${}_{2}$O${}_{3}$-nanoparticles.

**Figure 7:**
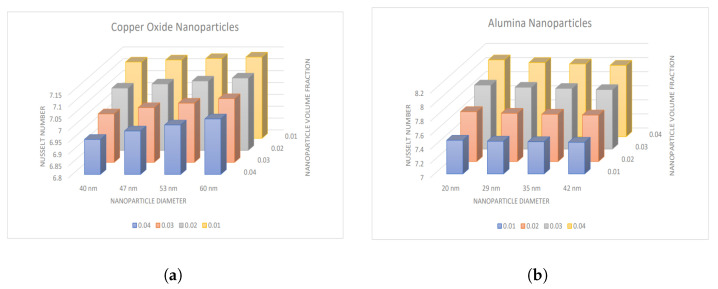
Variation in Nusselt number with nanoparticle diameter and volume fraction for (**a**) CuO-nanoparticles, (**b**) Al${}_{2}$O${}_{3}$-nanoparticles.

The impact of nanoparticle diameter and volume fraction for CuO and Al${}_{2}$O${}_{3}$-nanoparticles on temperature and Nusselt number is represented by Figure 6 and Figure 7. There is a decrement in the temperature values with increasing volume fraction of CuO nanoparticles, whereas an increment is observed with the diameter of nanoparticles. In contrast, an opposite trend for Al${}_{2}$O${}_{3}$-nanoparticles is analyzed, i.e., temperature boosts with increment in nanoparticle volume fraction and shows declination with enhancement in the diameter of nanoparticles. Also, the Nusselt number depicts the same variation in both cases, as shown in Figure 7a,b.

### 4.3. Velocity Contours

**Figure 8:**
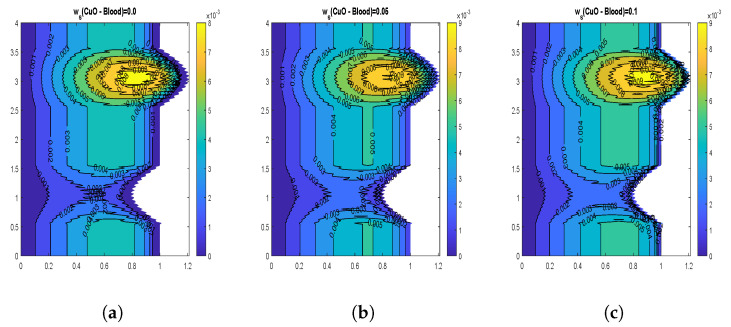
Blood flow patterns for wall slip velocity (**a**) $w_{s}=0.0$, (**b**) $w_{s}=0.05$, (**c**) $w_{s}=0.1$ for CuO-Blood.

**Figure 9:**
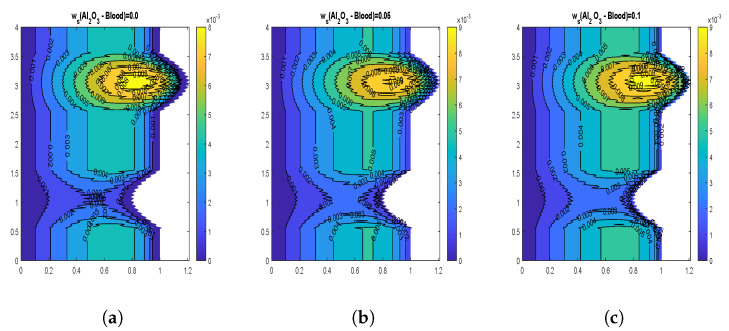
Blood flow patterns for wall slip velocity (**a**) $w_{s}=0.0$, (**b**) $w_{s}=0.05$, (**c**) $w_{s}=0.1$ for Al${}_{2}$O${}_{3}$-Blood.

**Figure 10:**
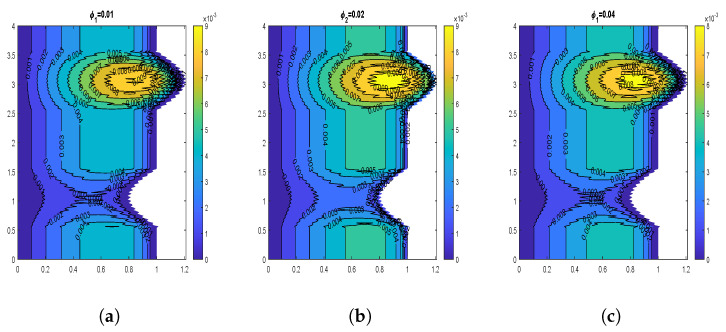
Blood flow patterns for volume fraction of CuO-nanoparticles (**a**) $\phi_{1}=0.01$, (**b**) $\phi_{1}=0.02$, (**c**) $\phi_{1}=0.04$.

**Figure 11:**
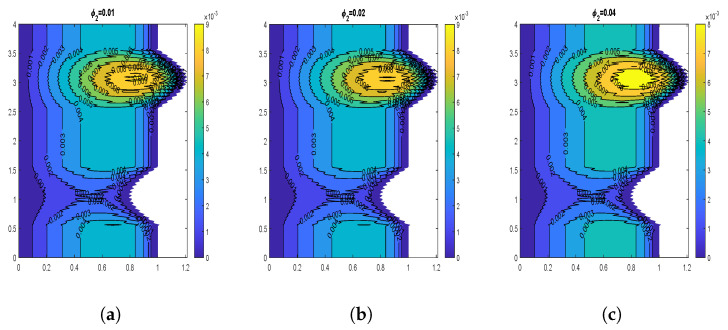
Blood flow patterns for volume fraction of Al${}_{2}$O${}_{3}$-nanoparticles (**a**) $\phi_{2}=0.01$, (**b**) $\phi_{2}=0.02$, (**c**) $\phi_{2}=0.04$.

The velocity contours for wall slip velocity and nanoparticle volume fraction are illustrated by Figure 8, Figure 9, Figure 10 and Figure 11. It is examined that the velocity rises as the $w_{s}$ values rise. No-slip velocity is typically considered at the artery wall, which has the least importance. As a result, the boundary conditions in the current investigation have been used to introduce wall slip at the artery wall by assuming the walls to be permeable. The area for maximum velocity first reduces as the $w_{s}$ value rises from $w_{s}$ = 0 to $w_{s}$ = 0.05, then expands as the value rises to $w_{s}$ = 0.1. Figure 8 and Figure 9 reveal that a similar behavior is noticed for both CuO and Al${}_{2}$O${}_{3}$ nanoparticles. The velocity shows declination with increasing volume fraction of both CuO and Al${}_{2}$O${}_{3}$ nanoparticles as depicted by Figure 10 and Figure 11. In the case of CuO nanoparticles, the maximum velocity region grows as the nanoparticle volume fraction reaches its maximum value. Additionally, there are more trapped bolus. In contrast, for Al${}_{2}$O${}_{3}$ nanoparticles, the maximum velocity region first exhibits a drop and subsequently increases. Additionally, there are fewer trapped bolus.

### 4.4. Impact of Electric Field Parameter

**Figure 12:**
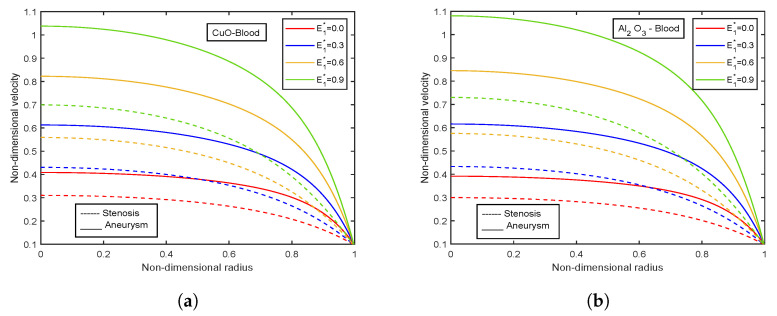
Velocity profiles of electric field parameter $E_{1}^{*}$ for (**a**) CuO-nanoparticles, (**b**) Al${}_{2}$O${}_{3}$-nanoparticles.

**Figure 13:**
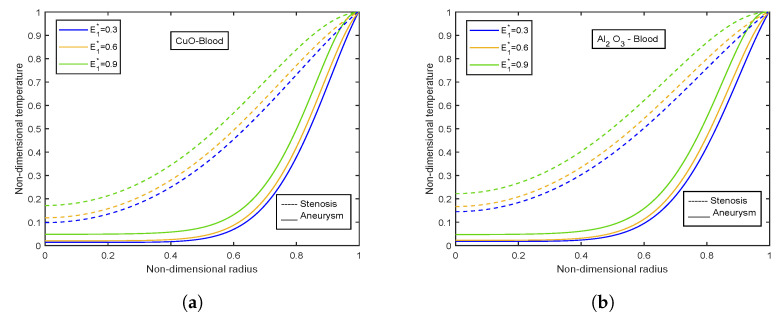
Temperature profiles of electric field parameter $E_{1}^{*}$ for (**a**) CuO-nanoparticles, (**b**) Al${}_{2}$O${}_{3}$-nanoparticles.

**Figure 14:**
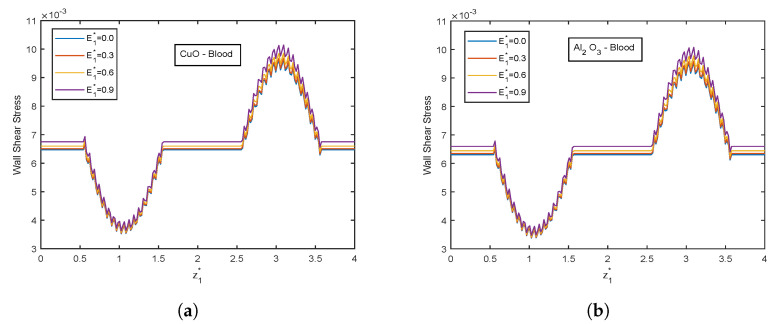
Wall shear stress profiles of electric field parameter $E_{1}^{*}$ for (**a**) CuO-nanoparticles, (**b**) Al${}_{2}$O${}_{3}$-nanoparticles at $t_{1}^{*}$ = 1.2.

**Figure 15:**
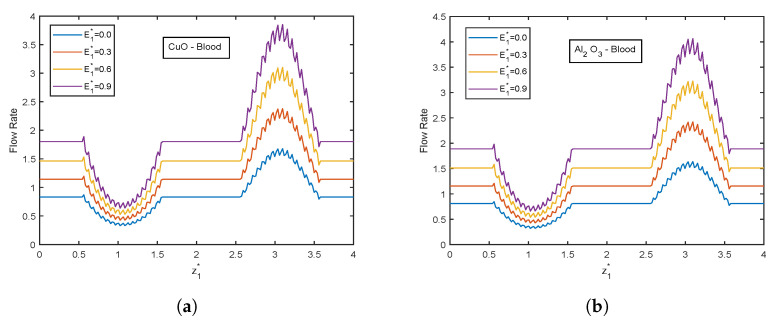
Flow rate profiles of electric field parameter $E_{1}^{*}$ for (**a**) CuO-nanoparticles, (**b**) Al${}_{2}$O${}_{3}$-nanoparticles at $t_{1}^{*}$ = 1.2.

**Figure 16:**
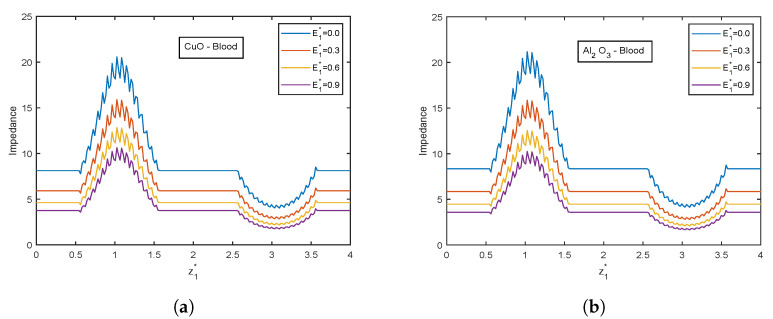
Impedance profiles of electric field parameter $E_{1}^{*}$ for (**a**) CuO-nanoparticles, (**b**) Al${}_{2}$O${}_{3}$-nanoparticles at $t_{1}^{*}$ = 1.2.

The velocity and temperature profiles for the electric field parameter are shown in Figure 12 and Figure 13. With higher electric field parameter $E_{1}^{*}$, there is a rising tendency for flow velocities and temperature. The electric field adds an accelerating force in the direction of the applied electric field, which causes the thickness of the momentum boundary layer to increase and causes the fluid to accelerate. Increased fluid velocity also causes a rise in velocity gradients, which in turn causes an increase in viscous dissipations. Hence, an increment in the temperature profiles. Figure 14 represents wall shear stress profiles for different values of $E_{1}^{*}$. The profiles show an increasing trend with $E_{1}^{*}$. The flow rate and impedance profiles for $E_{1}^{*}$ are depicted in Figure 15 and Figure 16. The flow rate increases with an increase in $E_{1}^{*}$ and, as expected, the opposite behavior for impedance profiles is observed.

### 4.5. Effect of Viscosity Parameter

**Figure 17:**
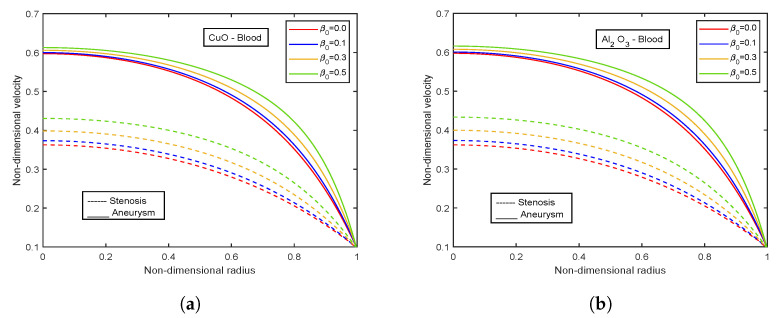
Impact of viscosity parameter $\beta_{0}$ on velocity profiles for (**a**) CuO-nanoparticles, (**b**) Al${}_{2}$O${}_{3}$-nanoparticles.

**Figure 18:**
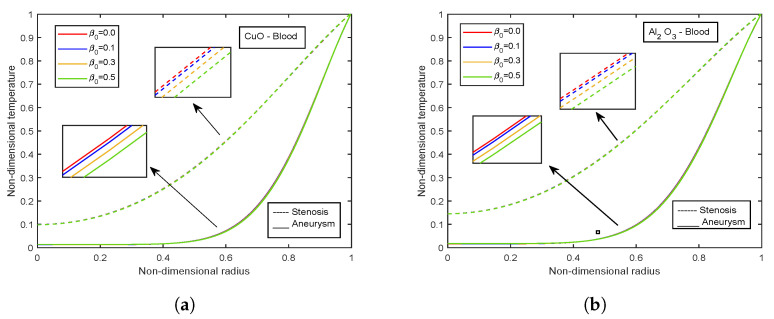
Impact of viscosity parameter $\beta_{0}$ on temperature profiles for (**a**) CuO-nanoparticles, (**b**) Al${}_{2}$O${}_{3}$-nanoparticles.

**Figure 19:**
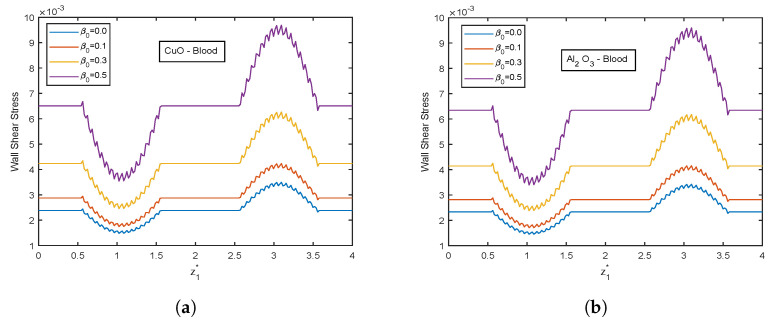
Wall shear stress profiles of viscosity parameter $\beta_{0}$ for (**a**) CuO-nanoparticles, (**b**) Al${}_{2}$O${}_{3}$-nanoparticles at $t_{1}^{*}$ = 1.2.

**Figure 20:**
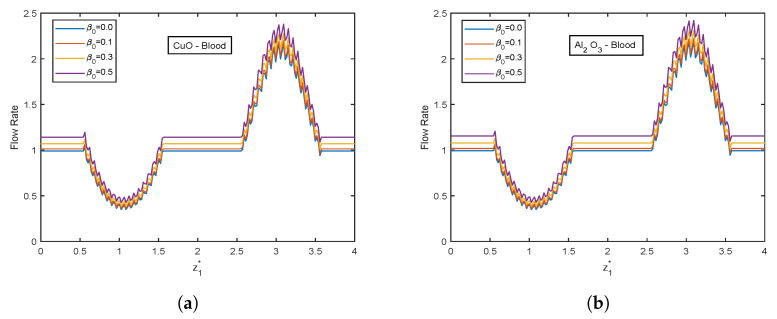
Flow rate profiles of viscosity parameter $\beta_{0}$ for (**a**) CuO-nanoparticles, (**b**) Al${}_{2}$O${}_{3}$-nanoparticles at $t_{1}^{*}$ = 1.2.

**Figure 21:**
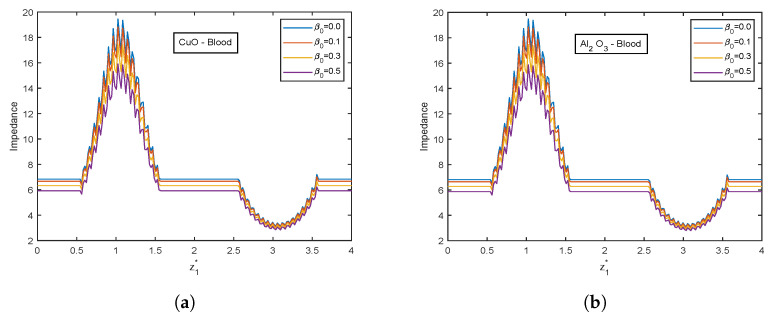
Impedance profiles of viscosity parameter $\beta_{0}$ for (**a**) CuO-nanoparticles, (**b**) Al${}_{2}$O${}_{3}$-nanoparticles at $t_{1}^{*}$ = 1.2.

The influence of viscosity parameter $\beta_{0}$ is highlighted in Figure 17, Figure 18, Figure 19, Figure 20 and Figure 21. The velocity profiles show inclination with increasing values of $\beta_{0}$ for both CuO and Al${}_{2}$O${}_{3}$ nanoparticles as conveyed by Figure 17a,b. This is because the resistance among the particles decreases when the magnitude of $\beta_{0}$ increases, indicating a rise in the velocity profiles. The temperature profiles show declination with enhancing $\beta_{0}$ values. An increment of 32.21% in the stenotic zone and 19.77% in the aneurysm zone is noticed when comparing the results for CuO and Al${}_{2}$O${}_{3}$ nanoparticles shown in Figure 18a,b. Figure 19 displays the profiles for wall shear stress for various $\beta_{0}$ values. As $\beta_{0}$ rises, the fluid becomes less viscous, enhancing the wall shear stress. Figure 20 and Figure 21 display flow rate and impedance profiles for various values of $\beta_{0}$. In the case of nanofluids, the flow rate rises as the value of $\beta_{0}$ rise, showing a viscosity reduction and leading to faster blood flow. Greater $\beta_{0}$ values represent a reduction in viscosity, which lowers impedance values.

### 4.6. Impact of Volume Fraction of Nanoparticles

**Figure 22:**
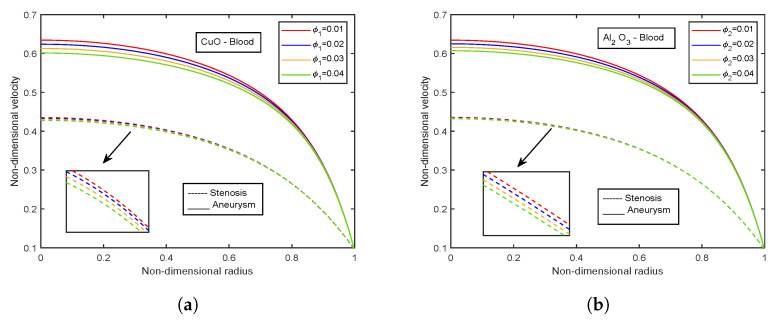
Influence of volume fraction of (**a**) CuO-nanoparticles, (**b**) Al${}_{2}$O${}_{3}$-nanoparticles on velocity profiles.

**Figure 23:**
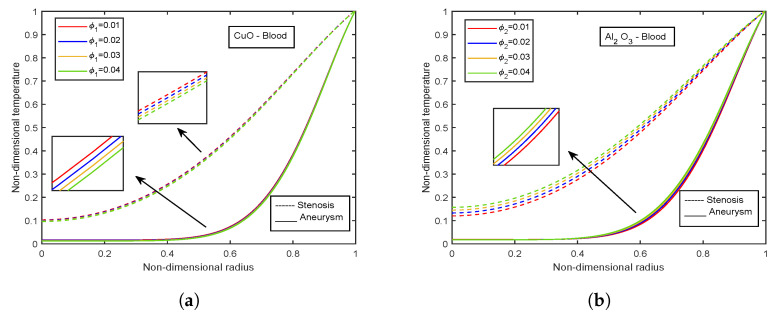
Influence of volume fraction of (**a**) CuO-nanoparticles, (**b**) Al${}_{2}$O${}_{3}$-nanoparticles on temperature profiles.

Figure 22 and Figure 23 depict the velocity and temperature profiles for different volume fractions of CuO-nanoparticles and Al${}_{2}$O${}_{3}$-nanoparticles, respectively. A higher volume proportion of nanoparticles reduces the fluid velocity. These flows are hindered by the growing magnetic viscosity that is related to the velocity dampening. Both the nanoparticles show a decreasing trend for velocity. However, Al${}_{2}$O${}_{3}$-nanoparticles observe slightly high-velocity values than CuO-nanoparticles of about 1.02% for the stenotic zone and 0.95% for the aneurysm region. Also, a comparison between stenotic and aneurysm regions reveals that velocity values in the aneurysm region are about 28.85% higher than that in the stenotic region. However, an opposite trend is observed for both the nanoparticle volume fractions in the case of temperature profiles. The temperature profiles decline with increasing volume fraction of CuO-nanoparticles, whereas an increment in temperature profiles is there with Al${}_{2}$O${}_{3}$-nanoparticles volume fraction. Also, the temperature values for the stenotic zone are higher than that of the aneurysm region.

### 4.7. Effect of Casson Fluid Parameter

**Figure 24:**
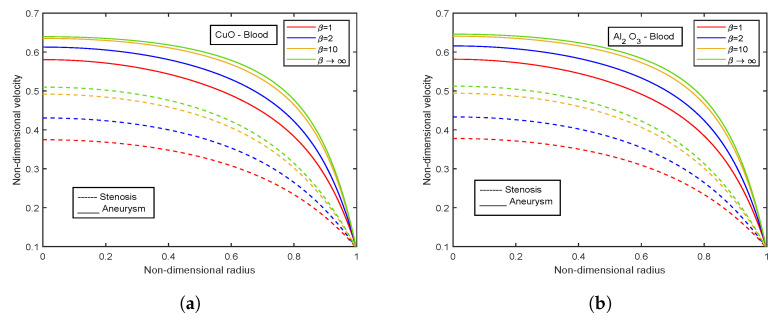
Velocity profiles of Casson fluid parameter β for (**a**) CuO-nanoparticles, (**b**) Al${}_{2}$O${}_{3}$-nanoparticles.

**Figure 25:**
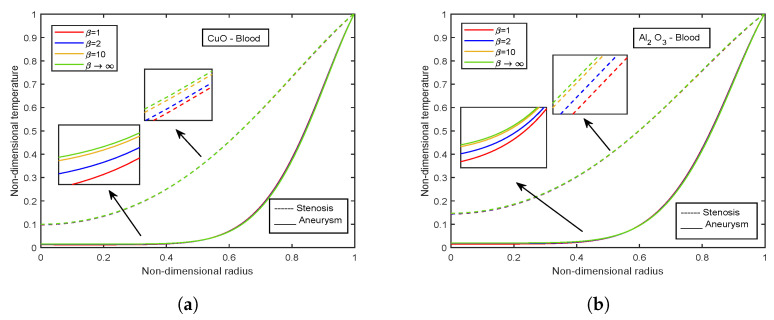
Temperature profiles of Casson fluid parameter β for (**a**) CuO-nanoparticles, (**b**) Al${}_{2}$O${}_{3}$-nanoparticles.

**Figure 26:**
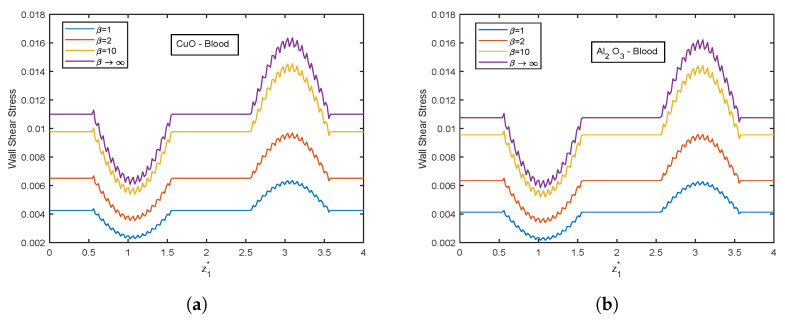
Wall shear stress profiles of Casson fluid parameter β for (**a**) CuO-nanoparticles, (**b**) Al${}_{2}$O${}_{3}$-nanoparticles.

**Figure 27:**
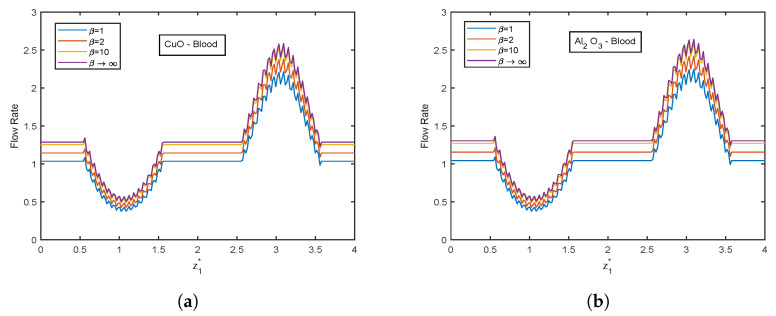
Flow rate profiles of Casson fluid parameter β for (**a**) CuO-nanoparticles, (**b**) Al${}_{2}$O${}_{3}$-nanoparticles at $t_{1}^{*}$ = 1.2.

**Figure 28:**
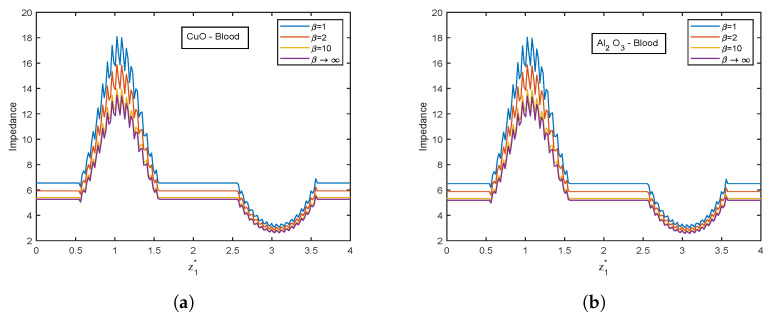
Impedance profiles of Casson fluid parameter β for (**a**) CuO-nanoparticles, (**b**) Al${}_{2}$O${}_{3}$-nanoparticles at $t_{1}^{*}$ = 1.2.

The influence of the Casson fluid parameter on the velocity and temperature profiles is depicted in Figure 24 and Figure 25. The Casson parameter varies inversely with the yield stress and is associated with the non-Newtonian Casson fluid character. Therefore, boosting β lowers the fluid’s yield stress, relaxing the fluid and allowing it to move more quickly. The velocity in the stenotic region is less than that in the aneurysm zone. The temperature profiles show an enhancement with increasing values of β. The Al${}_{2}$O${}_{3}$ nanofluid profiles are higher in magnitude by 30.96% than that of CuO- nanofluid. The stenotic zone has a higher temperature than the aneurysm zone. The wall shear stress profiles increase with rising β values, as illustrated in Figure 26. In the stenotic zone, CuO-nanofluid shows an increase of 4.18% over Al${}_{2}$O${}_{3}$-nanofluid. The flow rate profiles for β are displayed in Figure 27. The flow rate profiles follow the same trend as wall shear stress profiles. The fluid flows more easily as β increases because the yield stress of the fluid is reduced. As a result, there is less resistance in the fluid’s pathway, and impedance profiles decline as β enhances (Figure 28).

### 4.8. Effect of Radiation Parameter

**Figure 29:**
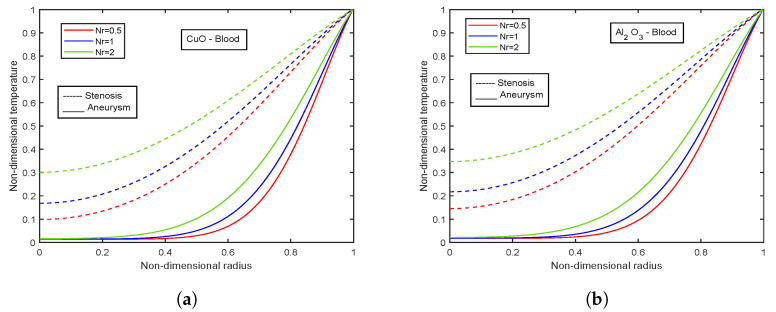
Influence of radiation parameter Nr on temperature profiles for (**a**) CuO-nanoparticles, (**b**) Al${}_{2}$O${}_{3}$-nanoparticles.

The temperature profiles of radiation parameter Nr for CuO and Al${}_{2}$O${}_{3}$ nanoparticles are displayed in Figure 29a,b. The system seems to radiate the most heat because Nr affects thermal conductivity in the opposite manner. Radiation acts as a heat source within the bloodstream, and a spike in radiation exposure causes body temperature to rise. Free electrons in nanoparticles oscillate when right-wavelength light interacts with them. These oscillations produce heat that spreads across the surrounding area and kills cancerous cells. The use of this discovery in thermal therapy is extensive. The temperature values are higher in the stenotic region than in the aneurysm region. Comparing the temperatures for CuO and Al${}_{2}$O${}_{3}$ nanoparticles reported an improvement of 13.28% in the stenotic zone and 24.85% in the aneurysm region.

### 4.9. Impact of Eckert Number

**Figure 30:**
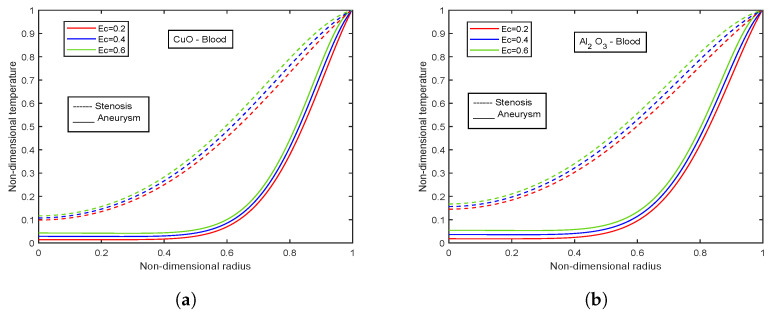
Temperature profiles of Eckert number Ec for (**a**) CuO-nanoparticles, (**b**) Al${}_{2}$O${}_{3}$-nanoparticles.

The temperature profiles for distinct values of Eckert number for CuO - Blood nanofluid and Al${}_{2}$O${}_{3}$-Blood nanofluid are illustrated in Figure 30a,b. The temperature profiles rise with increasing values of Ec. Additionally, compared to Al${}_{2}$O${}_{3}$-Blood nanofluid, CuO-Blood nanofluid has 30.6% greater magnitudes. The Eckert number connects the kinetic energy and enthalpy of a fluid. It’s a term to describe how a fluid is affected when it self-heats due to dissipation effects. The temperature profiles are affected by temperature gradients at high flow velocities and by dissipation effects resulting from internal friction between the fluid layers. Self-heating causes temperature profiles to rise, and these effects, in turn, impact the temperature profiles.

### 4.10. Effect of Prandtl Number

**Figure 31:**
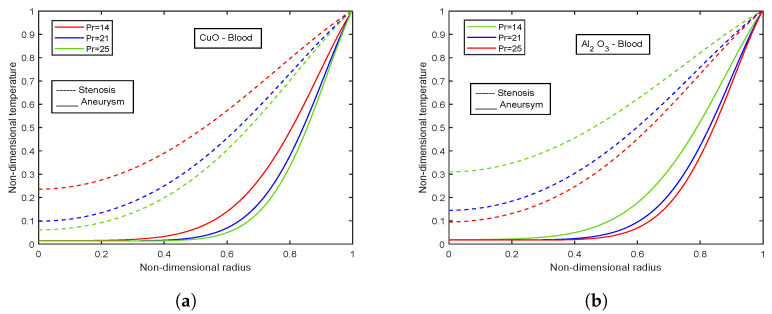
Impact of Prandtl number Pr on temperature profiles for (**a**) CuO-nanoparticles, (**b**) Al${}_{2}$O${}_{3}$-nanoparticles.

Figure 31a,b show the temperature profiles for different values of Prandtl number for CuO-Blood nanofluid and Al${}_{2}$O${}_{3}$-Blood nanofluid, respectively. An increase in Pr values accompanies the declination of the temperature profiles. The fluid’s viscosity and thermal diffusivity determine Pr. This dimensionless quantity links the momentum transport and the heat transport. The Prandtl number indicates whether heat is distributed more quickly in a fluid by convection or conduction. The Prandtl number has physical significance because, when it is less than 1, conductive heat transfer plays a significant role, transferring a higher percentage of heat than convection. Convective heat transfer takes precedence over conduction when the Prandtl value is higher than 1. A comparison between CuO-Blood nanofluid and Al${}_{2}$O${}_{3}$-Blood nanofluid reveals that Al${}_{2}$O${}_{3}$-Blood nanofluid reaches higher magnitudes for temperature by 23.94%. Also, the temperature values in the stenotic region are higher than in the aneurysm region.

## 5. Conclusions

The present model aims to simulate blood flow through a permeable stenosed artery with an aneurysm subject to the combined effect of electric and magnetic field (EMHD) using two types of nanoparticles, namely CuO and Al${}_{2}$O${}_{3}$ considering blood as Casson fluid. The viscosity and thermal conductivity have been modeled using KKL correlations. The effect of Joule heating, radiation, and viscous dissipation is considered. The governing equations have been solved using the Crank-Nicolson scheme. The findings have been validated and are in good agreement with those in the literature. The relative % variation in the Nusselt number has been calculated. Some of the significant conclusions of the current study are listed below:

- The temperature and Nusselt number boost with enhancing diameter of CuO nanoparticles and decreasing diameter of Al${}_{2}$O${}_{3}$ nanoparticles.
- Velocity and temperature profiles show inclination with increasing electric field parameter $E_{1}^{*}$.
- There is an escalation in velocity and wall shear stress profiles with viscosity parameter $\beta_{0}$.
- Flow rate ascends with increment in Casson fluid parameter β.
- Velocity descends with rising volume fraction of both CuO and Al${}_{2}$O${}_{3}$ nanoparticles.
- Temperature profiles elevate with volume fraction of Al${}_{2}$O${}_{3}$ nanoparticles, whereas declination is observed for CuO nanoparticles volume fraction.
- With increasing magnetic number, the relative % variation in the Nusselt number rises, but a decreasing trend is analyzed for the electric field parameter.

The application of nanotechnology in biomedicine is a fast-expanding subject with tremendous promises for enhancing the diagnosis and treatment of human disease. The ability to combine pharmaceuticals into a functionalized nanoparticle represents a new beginning for the selective delivery of therapeutics to tissues or cells. The present study’s findings may be helpful in the diagnosis of hemodynamic abnormalities and the fields of nano-hemodynamics, nano-pharmacology, drug delivery, tissue regeneration, wound healing, and blood purification systems.

## Figures and Tables

**Table 1:** Thermophysical properties of nanoparticles ([43]).

Thermophysical Properties	Blood	CuO	Al${}_{2}$O${}_{3}$
Density [ρ(kg/m${}^{3}$)]	1060	6500	3970
Thermal Conductivity [K(W/mK)]	0.492	18	25
Electrical Conductivity [σ(S/m)]	0.667	1 $\times \;10^{-10}$	3.5 $\times \;10^{7}$
Thermal Expansion Coefficient [$\gamma \times 10^{-5}$(K${}^{-1})$]	0.18	0.5	0.85
Heat Capacitance [$C_{p}$(J/kgK)]	3770	540	765
$d_{p}$	–	47	29

**Table 2:** The coefficient values of CuO and Al${}_{2}$O${}_{3}$ nanoparticles ([43]).

Coefficient	CuO	Al${}_{2}$O${}_{3}$
$b_{1}$	−26.593310846	52.813488759
$b_{2}$	−0.403818333	6.115637295
$b_{3}$	−33.3516805	1 0.6955745084
$b_{4}$	−1.915825591	4.17455552786 × $10^{-2}$
$b_{5}$	6.42185846658 × $10^{-2}$	0.176919300241
$b_{6}$	48.40336955	−298.19819084
$b_{7}$	−9.787756683	−34.532716906
$b_{8}$	190.245610009	−3.9225289283
$b_{9}$	10.9285386565	−0.2354329626
$b_{10}$	−0.72009983664	−0.999063481

**Table 3:** Grid Independence.

Grid Size	Wall Shear Stress ($\tau_{w}$)
25 × 25	0.0082
50 × 50	0.0035
70 × 70	0.0022
100 × 100	0.0018
200 × 200	0.0018

**Table 4:** Time Independence.

Time Step Size (dt)	Wall Shear Stress ($\tau_{w}$)
0.1	0.0035
0.08	0.0038
0.05	0.0042
0.02	0.0045
0.01	0.0044

**Table 5:** Default Values of emerging parameters ([48]).

Parameters	$\phi_{1}$	$\phi_{2}$	d	$B_{1}$	$c_{1}$	e	δ	$\beta_{0}$	$w_{s}$	β	α
Value	0.03	0.03	0.56	1.41	1	0.2	0.1	0.5	0.1	2	0.1

**Table 6:** Range of influential flow parameters.

Flow Parameter	Range	References
Magnetic Field Parameter (M${}^{2}$)	0–5	[37]
Casson fluid Parameter (β)	1–10	[29]
Grashof number (Gr)	0–0.5	[34,36]
Eckert number (Ec)	0–1	[36]
Prandtl number (Pr)	14–25	[46]
Radiation Parameter (Nr)	0–2	[18,25]

## Data Availability

Not applicable.

## Nomenclature

$r_{1}^{*}$	Radial direction
Ec	Eckert Number
$z_{1}^{*}$	Axial direction
Pr	Prandtl Number
$t_{1}^{*}$	Time
Nr	Radiation parameter
$u_{1}^{*}$	Velocity component in radial direction
Nu	Nusselt number
$w_{1}^{*}$	Velocity component in axial direction
Da	Darcy number
$U_{0}$	Reference velocity
Re	Reynold’s Number
R	Radius of artery in stenotic/aneurysm region
Gr	Grashof Number
$R_{0}$	Radius of artery in non-stenotic region
$Q_{1}$	Flow Rate
g	Acceleration by virtue of gravity
***Greek Letters***	
${\tilde{T}}^{*}$	Temperature of the base fluid
δ	Stenotic depth/Aneurysm height
${\tilde{T}}_{1}^{*}$	Reference temperature
$\tilde{\theta }$	Non-dimensional temperature
${\tilde{T}}_{w}^{*}$	Temperature at the wall
ρ	Density
$B_{0}$	Uniform Magnetic Field
$\phi_{1}$	Volume fraction of CuO NPs
${\tilde{C}}_{p}^{*}$	Specific heat at constant pressure
$\phi_{2}$	Volume fraction of Al${}_{2}$O${}_{3}$ NPs
$E_{0}$	Uniform Electric field
$\beta_{0}$	Viscosity constant
$k_{f}$	Thermal conductivity
λ	Resistance Impedance
$p_{1}^{*}$	Pressure
$\mu_{f}$	Blood’s viscosity
$w_{s}$	Wall slip velocity
$\mu_{0}$	Reference viscosity
$B_{1}$	Pressure gradient parameter
d	Location of stenosis/aneurysm
σ	Electrical conductivity
$L_{0}$	Length of stenosis/aneurysm
$\tau_{w}$	Shear stress at the wall
L	Length of the artery
α	Slip parameter
$k_{1}^{*}$	Permeability of the medium
β	Casson fluid parameter
$M^{2}$	Magnetic Number
γ	Thermal expansion coefficient
